# Bioprospecting the antimicrobial, antibiofilm and antiproliferative activity of *Symplocos racemosa* Roxb. Bark phytoconstituents along with their biosafety evaluation and detection of antimicrobial components by GC-MS

**DOI:** 10.1186/s40360-020-00453-y

**Published:** 2020-11-17

**Authors:** Henna Sood, Yashwant Kumar, Vipan Kumar Gupta, Daljit Singh Arora

**Affiliations:** 1 Microbial Technology Laboratory, Department of Microbiology, Guru Nanak Dev University, Amritsar, 143005 India; 2 National Salmonella & Escherichia Centre and Diagnostic Reagents Laboratory, Central Research Institute, Kasauli, HP 173204 India; 3 Department of Veterinary Pathology, Dr. G.C.Negi College of Veterinary and Animal Sciences, CSK Himachal Pradesh KrishiVishvavidyalaya, Palampur, HP 176062 India

**Keywords:** Antimicrobial, Drug resistant strains, Acute oral toxicity, Antibiofilm, Cytotoxicity, *Symplocos racemosa*

## Abstract

**Background:**

Plants provide a ray of hope to combat the ever increasing antibiotic resistance and *Symplocos racemosa* is a valuable medicinal plant. The study focused on highlighting the importance of this plant’s phytoconstituents as potential source of novel antimicrobials against planktonic as well as biofilm forming microorganisms, along with their antiproliferative activity. The biosafety of the phytoconstituents was also established, followed by detection of probable antimicrobial components.

**Methods:**

The best organic extractant and major groups of phytoconstituents were tested for their antimicrobial activity against reference microbial strains and drug-resistant clinical isolates. The anti-proliferative potential of the most active group of phytoconstituents was evaluated against cancerous cell lines. The in vitro biosafety of phytoconstituents was evaluated by Ames and MTT assay, while in vivo biosafety of the most active phytoconstituents, i.e., flavonoids was determined by acute oral toxicity. Further, the probable antimicrobial components in the flavonoids were detected by TLC and GC-MS.

**Results:**

Ethyl acetate extract was the most effective among various organic extracts, whereas phytoconstituents such as flavonoids, cardiac glycosides, saponins, tannins, triterpenes and phytosterols were the major groups present, with flavonoids being the most potent antimicrobials. The phytoconstituents displayed a significant antibiofilm potential, as exhibited by inhibition of initial cell attachment, disruption of the pre-formed biofilms and reduced metabolic activity of biofilms. The phytoconstituents were significantly active against the drug-resistant strains of *E.coli*, MRSA and *Salmonella* spp. Further, flavonoids showed significant cytotoxic effect against the cancerous cell lines but were non-cytotoxic against Vero (normal) cell line. All the test preparations were biosafe, as depicted by the Ames test and MTT assay. Also, flavonoids did not induce any abnormality in body weight, clinical signs, biochemical parameters and organs’ histopathology of the Swiss albino mice during in vivo acute oral toxicity studies. The flavonoids were resolved into 4 bands (S1-S4), where S3 was the most active and its GC-MS analysis revealed the presence of a number of compounds, where Bicyclo [2.2.1]heptan-2-one,1,7,7-trimethyl-, (1S)- was the most abundant.

**Conclusions:**

These findings suggest that the phytoconstituents from *Symplocos racemosa* bark could act as potential source of antimicrobial as well as antiproliferative metabolites.

## Background

Antibiotics have revolutionized the medical field and have helped us to live a healthy life. However, incidences of multiple resistances in human pathogenic microorganisms are on the rise and the problem has now become a global concern. The scientists are studying every aspect of antibiotic resistance [1] to come forward with possible ways out.

To meet out this objective, scientists are searching for new antimicrobials from various sources, where natural products still remain as one of the best reservoir for new compounds. The use of medicinal herbs is an age-old tradition and the recent progress in modern therapeutics has stimulated for further exploitation [2]. Numerous studies have revealed the potential of herbs as sources of drugs and have subsequently identified natural plant-based antimicrobial compounds [3–6]. The activity of these plants against different bacteria, fungi and parasites might be due to the presence of a wide variety of active secondary metabolites such as flavonoids, phenolic acids, coumarins, terpenoids and sterols [7–11].

In addition to being a potential source of novel antimicrobials, plants also play an important role in chemotherapy and represent a promising source of anticancer agents. However, the use of herbal products should be based on scientific validation‚ as the traditional use of any plant for medicinal purposes, by no means, guarantees the safety of the plant or treatment. Toxicity testing can identify any such risk that may be associated with herbal usage, therefore avoiding their potential short term or long term negative effects‚ when used as medicine [12].

One such potential medicinal plant is *Symplocos racemosa* Roxb. (also known as Lodhra) from the family Symplocaceae, which is an evergreen tree or shrub found in the plains and lower hills throughout North and East India [13]. Traditionally, bark is given in menorrhagia and other uterine disorders. It is a potent remedy for inflammation and cleaning uterus [14]. Since the potential of its aqueous extract has been reported earlier in Sood et al. [15], the present study has thus been further extended to evaluate the best organic solvent and hence the major groups of bioactive phytoconstituents. The organic extract and active phytoconstituents have been tested for their various bioactivities such as antimicrobial [Post Antibiotic Effect, Minimum Inhibitory Concentration studies and Time Kill Assay; antimicrobial efficacy against drug resistant clinical isolates; anti-biofilm activity] and anti-proliferative action against the cancerous cell lines (RD, L20B, Hep2) and normal cell line (Vero). The biosafety of all the test preparations was evaluated by Ames test and MTT assay. The in vivo biosafety of the flavonoids [at a single dose (5000 mg/kg)] has also been determined by acute oral toxicity assay using 8–10 weeks old Swiss albino mice (males and females) (Body weight: 25 g to 35 g) [12, 16–18].

## Methods

### Plant material

The plant *Symplocos racemosa* bark was procured from a local pansari market of Ram Bagh, near Hall Gate, Amritsar, Punjab (India); which has been identified and submitted to the Herbarium of the Department of Botanical and Environmental Sciences, Guru Nanak Dev University (Amritsar), India, with an accession no. 766–768 Bot.& Env. Sc. dated 02/07/15. Surface sterilization of the plant material was performed as described earlier in Sood et al. [15].

### Test microorganisms and inoculum preparation

All 12 reference strains of bacteria and yeast used in the study were obtained from Microbial Type Culture Collection (MTCC) of Institute of Microbial Technology (IMTECH), Chandigarh (India). A drug resistant isolate of MRSA‚ obtained from Post-Graduate Institute of Medical Education and Research (PGIMER), Chandigarh, India was also used in the study. All these cultures were preserved in the glycerol stock at − 80 °C.

To add upon value to the study, some drug resistant clinical isolates were also used, which included: MRSA (DSECI 1–11), *Enterococcus* sp. (DSECI 12) procured from Shri Guru Ram Das medical College & Hospital, Amritsar (Punjab), India; MDR *Escherichia coli* (CRIRS 1–12) and MDR *Salmonella* spp. (CRIRS 13–22) [obtained from Central Research Institute, Kasauli (H.P.)], India.

The inoculum of these organisms was prepared by 4 h activation in a suitable broth‚ followed by its standardization upto 0.5 McFarland standards [15].

### Organic extracts’ preparation and their antimicrobial screening

Different organic solvents such as ethyl acetate, chloroform, butanol, hexane and methanol were screened to work out the best extractant. The organic extracts were prepared by using solvent-solvent extraction of the aqueous extract (detailed in Additional file 1). The aqueous extract was prepared by suspending powdered plant material (17.5 g) in sterile distilled water (100 mL) and keeping it in hot water bath at 40 °C for 20 min. Hundred milliliter of filtered aqueous extract was then shaken vigorously in a separating funnel with an equal volume of a particular organic solvent, for three independent times. The pooled organic layers were concentrated in a rotavapor at 45 °C (under vacuum). The leftover material thus obtained were dissolved in diluted Dimethyl Sulfoxide (DMSO) [(30% (v/v)], which was then tested for their antimicrobial activity against the reference strains using the Agar Diffusion Assay (ADA).

The ADA studies were carried out as detailed in Additional file 1. The organic extract (0.1 mL of Ethyl acetate extract: 30.5 mg/mL; Butanol extract: 35.42 mg/mL; Hexane extract: 27.98 mg/mL; Chloroform extract: 26.44 mg/mL) was added to the wells and the plates were incubated in an upright position at 37 °C (bacterial cultures) and 25 °C (yeast strains) for 18–24 h. The antimicrobial activity could be observed as a clear zone of inhibition around the wells. Any organism with inhibition zone < 12 mm was considered as resistant to the extract. Here, 30% DMSO served as a negative control and the experiment was performed in duplicate.

### Phytochemical analysis of *Symplocos racemosa*

Qualitative analysis of the major groups of phytoconstituents (such as flavonoids, alkaloids, terpenoids, saponins, tannins, glycosides etc) in the powdered plant material was performed as per the standard methodology mentioned in Additional file 1. The qualitatively detected phytoconstituents were then quantified as described previously [19, 20] and screened for their antimicrobial potential along with some antibiotics (Additional file 1).

### Minimum Inhibitory Concentration (MIC) studies

The test preparations (ethyl acetate extract, flavonoids, cardiac glycosides) were studied for their MIC against the reference microbial strains (sensitive to the respective preparations in ADA) by Agar dilution method, as per the protocol followed in Arora and Sood [21] using various concentrations (Additional file 1) prepared from their respective stock solutions [i.e., Ethyl acetate extract: 30.5 mg/mL; Flavonoids: 54.75 mg/mL; Cardiac glycosides: 59.5 mg/mL]. Hundred microliters (100 μL) of the activated test organism (adjusted to 0.5 McFarland standards) was swabbed onto the solidified plates (containing different concentrations of a test preparation) and incubated at 37 °C (bacterial cultures) and 25 °C (yeast strains) for 18–24 h. The lowest concentration of the test preparations which inhibited the microbial growth were taken as their MIC. The values were compared with that of the standard antibiotics.

### Time kill assay and Post Antibiotic Effect (PAE)

In order to assess the microbistatic or microbicidal nature, time kill assay of the test preparations and standard antibiotics (Gentamicin and Amphotericin B) was performed [22] at their respective stock concentrations as mentioned above. The test preparation was mixed with the 10^− 3^ diluted inoculum of the test organism in the ratio 1:1. The inoculum containing only diluent was used as control. The mixture was then incubated at 37 °C (bacterial cultures) and 25 °C (yeast strains). The 100 μL aliquot was plated on respective medium every 2 h upto 24 h. The mean number of colonies was counted for both test and control group and the % cell viability was calculated.

The PAE for the above mentioned test preparations were worked out according to Raja et al. [23]. The 4 h activated test organism was diluted 1:1000 times using suitable broth. The diluted suspension of different organisms was mixed with the test preparations [as mentioned above] in an equal ratio in a test tube and incubated for 2 h at 37 °C (bacterial cultures) and 25 °C (yeast strains) under shaking condition. Simultaneously, a control containing the diluent (without test preparation) and the respective microorganism was also set up. Following incubation, the reaction mixture was serially diluted to 10^− 3^ using the suitable broth, whose 100 μL aliquot was withdrawn at every 2 h interval upto 24 h and plated onto the medium plates. After incubation of 24 h, the number of colonies was counted in the respective plates. The PAE was deduced using a formula, i.e., PAE = T – C (T = time taken for number of colonies in the test to increase by 1 log_10_CFU/mL; C = the time required in case of untreated control).

### Biosafety evaluation of *Symplocos racemosa* bark

#### Ames mutagenicity test

The Ames test for different test preparations (Ethyl acetate extract: 30.5 mg/mL; Flavonoids: 54.75 mg/mL; Cardiac glycosides: 59.5 mg/mL) was carried out by plate incorporation method, as described earlier [21] (for details see Additional file 1). Sodium azide was taken as a standard mutagen, while 30% DMSO were used as a negative control.

#### MTT [3-(4, 5-dimethylthiazol-2-yl)-2, 5-diphenyl tetrazolium bromide] assay

MTT assay was also done for the test preparations to find out their cytotoxic effect, as described earlier [24] with slight modifications (for details see Additional file 1). To calculate the % cell viability, the wells with untreated cells were used as control. Aliquots (100 μL) of blood cells’ suspension (in PBS; containing approximately 1 × 10^5^ cells/ mL) were dispensed into a 96 well microtitre plate in duplicates and after overnight incubation at 37 °C, the supernatant was removed carefully and 200 μL of the test preparations (as mentioned in above experiments) was added and incubated further for 1 day. Following incubation, the suspension was decanted off and the cells were exposed to 20 μL of MTT dye (0.5% w/v) at 37 °C/3.5 h, under mild shaking condition. The excess dye was pipetted out and DMSO (50 μL) was added for dissolving the end product (formazan), whose absorbance was then read at 590 nm in a 96 well-microtitre plate reader and the % viability of cells was calculated.

Since, flavonoids and cardiac glycosides showed comparatively better antimicrobial potential and were biosafe, thus, evaluated further for their antibiofilm potential and efficacy against drug resistant clinical isolates.

### Antibiofilm potential of the phytoconstituents of *Symplocos racemosa* bark

The antibiofilm potential was tested against four organisms, i.e., *Escherichia coli*, *Staphylococcus aureus*, *Klebsiella pneumoniae* 1 and a yeast *Candida albicans,* as described earlier [25–31] and detailed in Additional File 2.

#### Screening for biofilm formation and estimation of biofilm biomass by Crystal Violet (CV) assay

The biofilm formation by test pathogens was screened using microtitre plate according to Stepanovic et al. [25] and Costa et al. [26], where the organisms were grown in suitable broth for 24 h. The 200 μL aliquot of the activated cultures were dispensed into microtitre plates and further incubated at for 24 h, following which the wells were decanted off and stained with 0.1% crystal violet solution for 15 min. The wells were destained with 150 μL of 95% ethanol and the intensity of biofilm formation was affirmed in terms of Optical density (OD) of stained adherent biofilm by using an ELISA reader at 590 nm. The results were interpreted by calculating the cut-off value (ODc), which separates biofilm producers from the non-biofilm producers, as follows:

$$ \mathrm{Optical}\ \mathrm{density}\ \mathrm{cut}-\mathrm{off}\ \mathrm{value}\ \left(\mathrm{ODc}\right)=\mathrm{Average}\ \mathrm{OD}\ \mathrm{of}\ \mathrm{the}\ \mathrm{negative}\ \mathrm{control}+\left[3\times \mathrm{standard}\ \mathrm{deviation}\ \left(\mathrm{SD}\right)\ \mathrm{of}\ \mathrm{negative}\ \mathrm{control}\right]. $$

For the estimation of biofilm biomass in the succeeding assays, following the treatment of the test organisms, the culture medium from each well was discarded and plates were then washed with sterile distilled water and then dried. The plates were then stained for 15 min with a crystal violet solution (0.1% w/v), followed by washings with sterile distilled water. Absolute ethanol (125 μL) was then added to destain the wells and the absorbance was determined at 590 nm to quantitatively estimate the biofilm density and the percentage inhibition was calculated using the formula as described earlier [27–29]:

$$ \mathrm{Percentage}\ \mathrm{inhibition}=100-\left[\left\{{\mathrm{OD}}_{590\mathrm{nm}}\ \mathrm{test}\ \mathrm{preparation}\ \mathrm{well}/{\mathrm{OD}}_{590\mathrm{nm}}\ \mathrm{negative}\ \mathrm{control}\ \mathrm{well}\ \mathrm{without}\ \mathrm{test}\ \mathrm{preparation}\right\}\times 100\right]. $$

Upon confirmation‚ the following assays were carried out to establish the antibiofilm potential of the most active phytoconstituents.

#### Inhibition of initial cell attachment

The inhibitory potential of the partially purified phytoconstituents was carried out according to Jadhav et al. [30] and Onsare and Arora [27]. One hundred microlitres of flavonoids (54.75 mg/mL) and cardiac glycosides (59.5 mg/mL) were added to the 96-well microtitre plates, to which an equal volume of the cultures was then added. The plates were incubated at suitable temperature for 24 h. Gentamicin and amphotericin B were used as positive control. Following incubation, the inhibition potential was established using crystal violet assay (as described above).

#### Screening of phytoconstituents for their disruptive potential of preformed biofilms

It was performed as described earlier [26, 27] with slight modifications. One hundred microliter aliquot of each of the 4 h activated cultures were dispensed into a 96-well microtitre plate and were incubated at 37 °C/24 h to allow biofilm formation and was treated with equal volume of the phytoconstituents, i.e., flavonoids (54.75 mg/mL) and cardiac glycosides (59.5 mg/mL). The plates were further incubated at suitable temperature and biomass content was estimated after 24 h by the crystal violet assay.

#### Estimation of metabolic activity by XTT assay

Post exposure to the flavonoids, cardiac glycosides and standard antibiotics, the metabolic activity of the treated biofilms was assessed using the modified {2, 3-bis [2-methyloxy-4-nitro-5-sulfophenyl]-2H-tetrazolium-5-carboxanilide} (XTT) reduction assay [27, 31]. After incubation of the biofilm with the test preparations for 24 h, menadione–XTT mixture was then added to each well, followed by 2 h incubation at the suitable temperature (under dark conditions). The quantification of color was then done using microtitre plate reader (Bio-Rad 680-XR, Tokyo, Japan) at 490 nm. The mean absorbance of test wells was determined in comparison to that of negative control.

### Antimicrobial potential of the phytoconstituents against drug- resistant clinical isolates of MRSA, *Escherichia coli, Enterococcus* sp. and *Salmonella* spp.

The flavonoids (54.75 mg/mL) and cardiac glycosides (59.5 mg/mL) were also tested for their antimicrobial efficacy against clinical isolates of drug resistant strains (Table T1 of Additional File 5) of *Escherichia coli*, *Salmonella* spp., MRSA and *Enterococcus* sp. by Agar Diffusion Assay (Additional file 2). The sensitivity of different organisms was measured in terms of inhibition zone (in mm). The MIC of the most sensitive organisms was also worked out by broth dilution method [32]. Different concentrations of each phytoconstituent (0.09 mg/mL-50 mg/mL) were made from their respective stock and mixed with 50 μL of actively growing bacterial suspension in a 96 well microtitre plate. The inoculum without the test compound served as a negative control and the plates were incubated at 37 °C for 24 h. The lowest concentration with no visible growth was defined as the Minimum Inhibitory Concentration (MIC). To determine the MBC, the aliquots from the wells showing no visible growth were swabbed further onto the nutrient agar plates and incubated at 37 °C for 24 h. The concentration corresponding to no growth on the plates was taken as MBC.

The above experiments revealed the flavonoids to possess a better and significant bioactivity in comparison to cardiac glycosides, hence, were pursued further to evaluate their anti-proliferative potential.

### In vitro cytotoxicity against cancerous cell lines, i.e., RD (human Rhabdomyosarcoma), Hep2 (human epithelioma of larynx) and L20B (diploid mouse lung cell line)

The flavonoids was studied for their activity against the three cell lines obtained from Central Research Institute (C.R.I), Kasauli (H.P.), India by MTT [3-(4,5-dimethylthiazol-2-yl)-2,5-diphenyl tetrazolium bromide] assay as described previously [33–35] with slight modifications. The cytotoxic effect was compared to a normal cell line*,* i.e., Vero (African Green monkey kidney normal cell) line. The experiment was performed in triplicates as per protocol detailed in Additional file 3. Two fold serial dilutions (10 mg/mL to 0.039 mg/mL) were prepared from a stock (10 mg/mL), where the untreated cells (containing only diluent, i.e., 30% DMSO) were taken as control. The percent growth inhibition was calculated with respect to untreated control. The IC_50_ of the flavonoids against the tested cancerous cell lines was deduced from the resultant graphs obtained.

The flavonoids were the most effective phytoconstituents, as evident from their significant antimicrobial, antibiofilm and antiproferative potential and were biosafe as revealed by in vitro assays. Therefore, to re-ensure their biosafety and consumability, they were subjected to in vivo acute oral toxicity study.

### In vivo acute oral toxicity studies of flavonoids

The in vitro biosafety of the flavonoids was re-confirmed under in vivo conditions using an animal model (Swiss albino mice), as per the experimental details mentioned earlier [12, 16], with slight modifications, which has been detailed in Additional file 4. Total 24 healthy Swiss albino mice (males and females) (25–35 g body weight and 8–10 weeks old), were procured through proper channel from the Animal Breeding Facility of the Animal house of Central Research Institute, Kasauli, Himachal Pradesh, India. The mice were randomly divided into 2 test groups (6- male; 6- female) and 2 control groups (6- male; 6- female), where the test groups were exposed to a single dose (5000 mg/kg) of the flavonoids by oral route. This study was carried out in accordance with the principles of the Basel Declaration and recommendations of Organization of Economic Co-operation and Development (OECD) guideline 420. The study was carried out at Central Research Institute, Kasauli and the protocol was approved by the Institutional Animal Ethics Committee (IAEC) of Central Research Institute (C.R.I), Kasauli (H.P.), India; working under the guidance of Committee for the Purpose of Control and Supervision of Experiments on Animals (CPCSEA), New Delhi, India (No. CPCSEA/IAEC/CRI/14–114-2016). On the 15th day, the final weight of mice was noted and were anesthetized in the laboratory using a mixture of xylaxine and ketamine (at a concentration of 5 mg/kg b.wt. and 2.5 mg/kg b.wt., respectively). The blood samples were collected and the separated serum was used to determine the Liver functioning (Bilirubin content, ALT, AST, ALP) as well as Kidney functioning parameters (Creatinine, Urea). All the unconscious animals were then subjected to euthanasia by an overdose of anesthesia (mixture of xylaxine and ketamine at a concentration of 5 mg/kg and 2.5 mg/kg b.wt., respectively), as anesthetic overdose is the most commonly used method for euthanasia of mice because of being a simple, aesthetic and least painful method, with a lower blood loss associated with euthanasia [36]. Following euthanasia, the liver, kidney and heart from all the mice were extracted and prepared for the histopathological analysis.

### Detection of the probable bioactive components of the flavonoids

In order to find out the component responsible for antimicrobial activity of the most active phytoconstituent, i.e.*,* flavonoids, various techniques such as TLC, Preparative-TLC and Gas Chromatography-Mass Spectrometry (GC-MS) analysis were performed.

#### Thin Layer Chromatography (TLC) analysis

To work out the best solvent system, the quantitatively isolated flavonoids were subjected to TLC on the pre-coated Silica gel F_254_ plates using different solvent systems. The developed chromatograms were allowed to air dry and were visualized using natural light, UV light (254 nm, 365 nm) and iodine vapors so as to assess the degree of separation of bands and the Retention factor (R_f_) were calculated for the bands resolved in the most suitable solvent system. The best worked out solvent system was taken up for quantitatively separating the bands using Preparative TLC (P-TLC) method.

#### Quantitative separation of the bands using Preparative TLC (P-TLC) method and their antimicrobial screening

The bands were resolved and separated on Silica gel F_254_ using the selected combination of extraction solvents. Once developed, the separated bands were scrapped off carefully into separate vials, which were eluted overnight in methanol. The solvent containing the eluted band was decanted off carefully, which was evaporated to dryness to obtain a constant weight. The separated bands were dissolved in a minimum known volume of methanol and were screened for their antimicrobial activity against *Staphylococcus aureus*, *Staphylococcus epidermidis* (Gram positive); *Klebsiella pneumoniae* 1, *Shigella flexneri* (Gram negative) and a yeast *Candida albicans* using Disc diffusion method, where the filter paper discs were impregnated with 20 μL of this suspension, while methanol alone acted as a negative control. The band/s showing the most significant antimicrobial activity was then subjected to GC-MS analysis.

#### Gas Chromatography-Mass Spectrometry (GC-MS) analysis

The GC-MS analysis of the most active band/s was carried out using Thermo Trace 1300GC coupled with Thermo TSQ 800 Triple Quadrupole MS with column BP 5MS (30 m X 0.25 mm, 0.25 μm). The instrument was set to an initial temperature of 60 °C and maintained at this temperature for 3 min. At the end of this period the oven temperature was raised to 280 °C, at an increase rate of 15 °C/ min and maintained for 19 min. Injection port temperature was ensured as 260 °C and Helium flow rate as 1.2 mL /min. The ionization voltage was 70 eV. The injector was used with S (split) mode, with a split ratio of 30:1 and the injection volume of samples was 1 μL (1 mg/mL). Mass spectral scan range was set at 50–650 (m/z). Using computer searches on a NIST Ver. 2.0 MS data library and by comparing the spectrum obtained through GC-MS, compounds probably present in the flavonoids were identified.

### Data analysis

The statistical analysis was done by One way ANOVA followed by Tukey’s t-test at 95% level of confidence using IBM SPSS Statistics Data editor Version 20 in case of acute oral toxicity study, antimicrobial activity of organic extracts and antimicrobial evaluation against MDR clinical isolates. Standard error of means (SEM) was applied to Table 2, 3, 4, 5 and 6 using Microsoft excel, 2010 and IC_50_ in the in vitro cytotoxicity assay was calculated using Microsoft excel, 2010.

## Results

### Organic extracts’ preparation and their antimicrobial screening

To find out the best organic solvent for effective leaching out of the active components, various solvents such as ethyl acetate, butanol, chloroform, hexane and methanol were used, where methanol was miscible with the aqueous extract of *Symplocos racemosa*. Among all the organic extracts tested, the ethyl acetate extract demonstrated the highest activity against 10 out of 13 test organisms, with an average inhibition zone (IZ) of 14.92 mm, where *Staphylococcus aureus* (Gram positive), *Klebsiella pneumoniae* 1 (Gram negative) and *Candida albicans* (Yeast) were the most sensitive organisms with an IZ of 19.5 mm, 23 mm and 21 mm, respectively (Fig. A6 in Additional File 5). Butanolic extract followed up next and was active against 9 out of 13 strains (average IZ = 10.92 mm against all the tested organisms). Here, *Staphylococcus aureus* was the most sensitive organism (19 mm). The hexane extract was active against *Candida albicans* and *Klebsiella pneumoniae* 1, while the chloroform extract was completely inactive against all the tested microorganisms. The efficacy of ethyl acetate extract and the butanolic extract did not differ significantly from each other (*p* > 0.05), but ethyl acetate had a higher average IZ against the test organisms, hence was considered as the best organic extractant. The efficacy of both the extracts differed significantly from that of hexane extract of *Symplocos racemosa* (*p* ≤ 0.05) at 5% level of significance, as revealed by One way ANOVA followed by Post hoc Tukey’s t-test. *Enterococcus faecalis*, *Klebsiella pneumoniae* 2 and *Candida tropicalis*, however, remained resistant to all the organic extracts of *Symplocos racemosa.*

### Qualitative and quantitative phytochemical evaluation

Major phytochemical groups such as flavonoids, cardiac glycosides, saponins, tannins, triterpenes and phytosterols were detected (Table 1); however, alkaloids, diterpenes and coumarins were absent. Among the isolated phytoconstituents, cardiac glycosides were the most abundant, i.e., 50.25%/g plant powder, followed by flavonoids (10.39% /g), while triterpenes (1.193% /g) were present in least amount (Fig. A1 in Additional file 5).

**Table 1:** Qualitative phytochemical analysis of *Symplocos racemosa* bark

Phytoconstituents	Detected group	Stock solution (mg/mL)	Antimicrobial activity
**Alkaloids**		**NA**	**NA**
Mayer’s reagent test	**-** ^**a**^		
Hager’s reagent test	**–**		
Wagner reagent test	**–**		
**Flavonoids**		**54.75**	**+++** ^**c**^
Shinoda test (Magnesium turnings)	**+**^**b**^		
Zinc-hydrochloride reduction test	**+**		
Lead acetate test	**+**		
Ferric chloride reagent test	**–**		
**Saponins**		**41.6**	**+** ^**d**^
Froth test	**+**		
**Tannins**		**100.5**	**+**
Ferric chloride reagent test	**+**		
Lead acetate test	**+**		
**Cardiac glycosides**		**59.5**	**++** ^**e**^
Keller- killiani test	**+**		
**Terpenoids**			
Triterpenes (Salkowski’s test)	**+**	**12**	**-** ^**f**^
Diterpenes (Copper acetate test)	**–**	**NA**	**NA**
**Anthranol glycosides**		**ND**	**ND**
Borntrager’s test	**+**		
**Phytosterols**		**38**	**–**
Libermann Burchard’s test	**+**		
Salkowski’s test	**+**		
**Coumarins**	**–**	**ND**	**–**

^a^- absent; ^b^- present; ^c^- most active; ^d^- least active; ^e^- active; ^f^- not active

*NA–* Not applicable, *ND–* Not done

Among all the isolated phytoconstituents, flavonoids were the most effective (Table 2) with inhibition zone ranging from 16.6 mm to 25.66 mm, but were ineffective against *Enterococcus faecalis*. However, *Candida albicans* (25.66 mm) and *Candida tropicalis* (17.3 mm) were quite sensitive to it. Cardiac glycosides showed an inhibition zone ranging from 12.3 mm to 25.66 mm, where *Klebsiella pneumoniae* 1 showed maximum susceptibility. *Salmonella* Typhimurium 1, *Escherichia coli*, *Shigella flexneri* and yeast *Candida tropicalis* were resistant to it. Saponins were effective only against *Klebsiella pneumoniae* 1 and *Candida albicans*. However, triterpenes, tannins and phytosterols were completely inactive against all the test microorganisms. The antimicrobial efficacy of flavonoids was comparable to both the antibiotics (gentamicin and chloramphenicol) in case of *Staphylococcus epidermidis,* whereas it was comparable to chloramphenicol in case of *Escherichia coli, Klebsiella pneumoniae* 2 and *Pseudomonas aeruginosa*.

**Table 2:** Antimicrobial activity of phytoconstituents isolated from *Symplocos racemosa* bark

Test Organisms^**b**^	Average zone of inhibition (mm)**				
	Flavonoids	Saponins	Cardiac Glycosides	Gentamicin^**¥**^	Chloramphenicol^**¥**^
**SA**	19.66 ± 0.333	-*	15.66 ± 0.333	34.5 ± 0.50	26 ± 1.00
**SE**	22.33 ± 0.333	–	16.33 ± 0.333	26.5 ± 0.50	28.5 ± 0.50
**EC**	23 ± 1	–	–	31 ± 1.00	25 ± 0
**EF**	–	–	12.33 ± 0.333	27.5 ± 0.50	26.5 ± 0.50
**KP1**	20.33 ± 0.333	14.33 ± 0.333	25.66 ± 0.333	40.5 ± 0.50	38 ± 1.00
**KP2**	25.66 ± 0.333	–	19.33 ± 0.333	37.5 ± 1.50	26.5 ± 0.50
**SF**	16.66 ± 0.333	–	–	30.5 ± 0.50	27.5 ± 0.50
**ST1**	16 ± 0	–	–	35 ± 0	23 ± 0
**ST2**	24 ± 0.577	–	17 ± 0	43 ± 1.00	40.5 ± 0.50
**PA**	21.66 ± 0.333	–	15.66 ± 0.333	40.5 ± 0.50	28.5 ± 0.50
**CA**	25.66 ± 0.333	15.33 ± 0.333	25 ± 0	36.5 ± 0.50^a^	ND
**CT**	17.33 ± 0.333	–	–	27.5 ± 0. 50^a^	ND
**MRSA**	24.33 ± 0.666	–	17.66 ±0.333	42 ± 0	39.5 ± 0.50

Concentrations used: Flavonoids- 54.75 mg/mL; Saponins- 41.6 mg/mL; Cardiac glycosides- 59.5 mg/mL; ^¥^Concentration used: 1 mg/mL

*No activity; ** Values are expressed as Mean ± SEM of three determinations; *ND–* not done

^a^Amphotericin B (1 mg/mL); ^b^ Organisms- SA- *Staphylococcus aureus* (MTCC 740); SE- *Staphylococcus epidermidis* (MTCC 435); EC- *Escherichia coli* (MTCC 119); EF- *Enterococcus faecalis* (MTCC 439); KP1- *Klebsiella pneumoniae* 1 (MTCC 109); KP2- *Klebsiella pneumoniae* 2 (MTCC 530); SF- *Shigella flexneri* (MTCC 1457); ST1- *Salmonella* Typhimurium 1 (MTCC 98); ST2- *Salmonella* Typhimurium 2 (MTCC 1251); PA- *Pseudomonas aeruginosa* (MTCC 741); CA- *Candida albicans* (MTCC 227); CT- *Candida tropicalis* (MTCC 230); MRSA- Methicillin- Resistant *Staphylococcus aureus*

### Minimum Inhibitory Concentration (MIC)

MIC of the ethyl acetate extract, flavonoids and cardiac glycosides was quite variable (Table 3). Ethyl acetate extract was found to be highly potent (with MIC range of 0.5–3 mg/mL), in comparison to the phytoconstituents (Flavonoids and Cardiac glycosides), with the MIC values being lowest against *Salmonella* Typhimurium 2 and *Pseudomonas aeruginosa*. Further, the MIC for cardiac glycosides and flavonoids ranged from 0.5–10 mg/mL and 0.7–10 mg/mL, respectively, where *Klebsiella pneumoniae* 1 and *Candida albicans* were the most sensitive organisms showing the lowest MIC values. For *Pseudomonas aeruginosa* and MRSA, cardiac glycosides showed a higher MIC (10 mg/mL and 5 mg/mL, respectively) than the ethyl acetate extract (0.5–0.7 mg/mL) and flavonoids (1 mg/mL). The MIC values obtained for ethyl acetate extract and flavonoids were comparable to that of chloramphenicol against *Pseudomonas aeruginosa.*

**Table 3:** Minimum Inhibitory Concentration (MIC) of the various test preparations of *Symplocos racemosa* bark

Organisms^**d**^	MIC (mg/mL)				
	EA ^**b**^	Flavonoids	Cardiac glycosides	Gentamicin	Chloramphenicol
**SA**	0.7	3	1	0.0002	0.01
**SE**	1	3	1	0.01	0.01
**EC**	1	3	ND	0.005	0.01
**EF**	ND^a^	ND	ND	0.03	0.3
**KP1**	0.5	0.7	0.5	0.0002	0.01
**KP2**	ND	1	10	0.0005	0.001
**SF**	1	3	ND	0.005	0.01
**ST1**	3	1	ND	0.005	0.1
**ST2**	0.5	3	3	0.0003	0.001
**PA**	0.5	0.7	10	0.005	0.7
**CA**	0.7	0.7	0.5	0.0003^c^	ND
**CT**	ND	10	ND	0.1^c^	ND
**MRSA**	1	1	5	0.005	0.01

^a^ Not determined; ^b^ Ethyl acetate extract; ^c^Amphotericin B^d^; SA- *Staphylococcus aureus*; SE- *Staphylococcus epidermidis*; EC- *Escherichia coli*; KP1- *Klebsiella pneumoniae* 1; KP2- *Klebsiella pneumoniae* 2; SF- *Shigella flexneri*; ST1- *Salmonella* Typhimurium 1; ST2- *Salmonella* Typhimurium 2; PA- *Pseudomonas aeruginosa*; CA- *Candida albicans*; CT- *Candida tropicalis*; MRSA- Methicillin- Resistant *Staphylococcus aureus*

### Time kill assay and Post Antibiotic Effect (PAE)

A variable killing time was observed for different test preparations (ethyl acetate extract, flavonoids and cardiac glycosides) against different test pathogens (Fig. A2 in Additional file 5). *Pseudomonas aeruginosa* and *Candida albicans* were killed instantaneously upon incubation with ethyl acetate extract, whereas *Salmonella* Typhimurium 1 took a maximum of 24 h incubation period to achieve complete killing. In case of *Shigella flexneri* and MRSA, the killing time (2 h and 6 h, respectively) was similar to that of standard antibiotic gentamicin. Flavonoids were also quite effective against the test organisms, where *Candida albicans* was killed instantaneously. *Escherichia coli* and *Pseudomonas aeruginosa* were killed completely in 2 h*,* whereas *Salmonella* Typhimurium 1 and *Salmonella* Typhimurium 2 took 6 h for complete killing. Their Killing time was lesser than Gentamicin in case of organisms like *Staphylococcus aureus*, *Klebsiella pneumoniae* 2, *Salmonella* Typhimurium 2, *Pseudomonas aeruginosa and Candida albicans.* Cardiac glycosides had a kill time falling between 2 and 8 h, where 2 h incubation was sufficient to kill *Klebsiella pneumoniae* 1 and *Candida albicans.* They were better in case of organisms like *Klebsiella pneumoniae* 1 and *Klebsiella pneumoniae* 2. Flavonoids were more efficient in killing majority of the organisms than the ethyl acetate extract and cardiac glycosides, as evident from their lesser killing time. All the test preparations (ethyl acetate extract, flavonoids and cardiac glycosides) were highly effective against *Escherichia coli* (2 h), *Pseudomonas aeruginosa* (0–4 h) and *Candida albicans* (0–2 h).

Further, Flavonoids and cardiac glycosides showed a Post Antibiotic Effect **(**PAE) of 2–4 h and 2–6 h, respectively (Fig. A3 in Additional file 5). Flavonoids were equally effective (4 h) against *Salmonella* Typhimurium 2 and *Klebsiella pneumoniae* 2, whereas cardiac glycosides had a maximum effect against *Candida albicans* (6 h). The ethyl acetate extract also showed a similar PAE of 2–6 h. Among all the test preparations (ethyl acetate extract, flavonoids and cardiac glycosides), the longest effectivity was observed against *Candida albicans* (4–6 h), whereas for organisms like *Staphylococcus aureus*, *Salmonella* Typhimurium 2 and *Klebsiella pneumoniae* 2, their effect lasted for 2–4 h (Fig. A3 in Additional file 5).

### Biosafety evaluation

All the test preparations, i.e., ethyl acetate extract, flavonoids and cardiac glycosides were found to be non-cytotoxic and non-mutagenic in nature, as assessed by Ames test and MTT assay, respectively (Table T2 in Additional file 5). In Ames test, no revertant colonies were seen upon exposure to the test preparations as compared to the numerous revertant colonies (856) obtained upon exposure to the known mutagen*,* i.e.*,* sodium azide. In MTT assay, the test preparations were found to be non-cytotoxic as evident from the high viability of the blood cells post-exposure [96.02% (ethyl acetate extract); 91.83% (flavonoids); 96.56% (cardiac glycosides)].

### Antibiofilm potential of the phytoconstituents isolated from *Symplocos racemosa* bark

The most active phytoconstituents, i.e., flavonoids and cardiac glycosides were assessed for their antibiofilm potential against the test pathogens. The Optical Density cut-off value (ODc) of the negative control was 0.097. All the test organisms were found to be strong biofilm-formers as their average OD values were higher than 4 × ODc (0.388), viz., 0.649 (*Klebsiella pneumoniae* 1), 0.720 (*Staphylococcus aureus*), 0.892 (*Candida albicans*) and 0.557 (*Escherichia coli*).

#### Inhibition of initial cell attachment

**Fig. 1:**
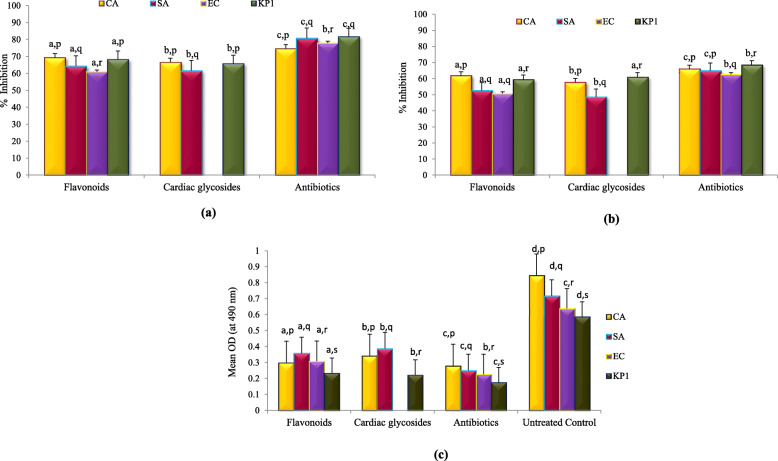
Inhibitory action of the Partially Purified Phytoconstituents of *Symplocos racemosa* bark on the **a** Initial cell attachment **b** Pre-formed biofilms of the test organisms **c** Metabolic activity of the biofilms.*EC-*Escherichia coli*; KP1- *Klebsiella pneumoniae* 1, CA- *Candida albicans,* SA- *Staphylococcus aureus*. Similar alphabetic letters on the bar columns before and after the comma represent no significant difference at 95% confidence level among different treatments for each organism and among test organisms for each treatment, respectively as indicated by One-way ANOVA followed by Tukey’s t-test

The flavonoids and cardiac glycosides effectively inhibited the attachment of the planktonic cells of these organisms (Fig.1a). Flavonoids (% Inhibition: 61.01–69.33%) were found to be more active than cardiac glycosides (% Inhibition: 61.64–66.66%) against the tested organisms. The maximum % inhibition was seen against *Candida albicans* and *Klebsiella pneumoniae* 1. The inhibitory potential of phytoconstituents was in close proximity to the respective antibiotics (gentamicin and amphotericin B) (Fig.1a). This inhibitory effect was statistically compared, where the inhibitory potential of the phytoconstituents and the standard antibiotics was statistically different (*p* ≤ 0.05) against all the four test organisms, as revealed by One way ANOVA followed by Tukey’s t-test. The individual effect of phytoconstituents and antibiotics on the four test organisms was statistically variable and significant, however, the inhibitory effect of flavonoids and cardiac glycosides against *C. albicans* and *K. pneumoniae* 1; antibiotic (gentamicin) against *S. aureus* and *K. pneumoniae* 1 did not show any statistical difference (*p* ≥ 0.05) (Fig. 1a).

#### Disruptive potential on the pre-formed biofilms

The test preparations exhibited varying efficacy towards the pre-formed biofilms of the test organisms (Fig. 1b). Flavonoids were more effective to disrupt the pre-formed biofilms than the cardiac glycosides. In case of *Candida albicans*, the inhibitory potential of flavonoids (61.85%) and cardiac glycosides (57.73%) was comparable to amphotericin B (65.97%), though the values showed statistically significant difference (p ≤ 0.05)*.* The inhibitory potential of the phytoconstituents and the standard antibiotics was statistically different (p ≤ 0.05) against all the four test organisms, as revealed by One way ANOVA followed by Tukey’s t-test, except for the inhibitory effect of flavonoids and cardiac glycosides against *K. pneumoniae* 1. The individual effect of phytoconstituents and antibiotics on the four test organisms was statistically variable and significant, however, the inhibitory effect of flavonoids against *S. aureus* and *E. coli*; antibiotic (gentamicin) against *S. aureus* and *C. albicans* did not show any statistical difference (p ≥ 0.05) (Fig. 1b).

#### Metabolic activity of the treated pre-formed biofilms by using XTT assay

The flavonoids and cardiac glycosides considerably reduced the cell viability, as indicated by the reduced metabolic activity (low mean absorbance values) (Fig. 1c). In case of *Candida albicans,* flavonoids (mean absorbance = 0.298) were more effective than cardiac glycosides (mean absorbance = 0.342), however, both of them had an effect comparable to amphotericin B (mean absorbance = 0.279). Cardiac glycosides reduced the metabolic activity of *Klebsiella pneumoniae* 1 biofilm more efficiently than the flavonoids, whereas, the effect of flavonoids and cardiac glycosides was almost similar in case of *Staphylococcus aureus*. These were found to be good biofilms inhibitors by efficiently reducing the metabolically active cells. It was observed that the inhibitory potential of the phytoconstituents and the standard antibiotics was statistically different (*p* ≤ 0.05) from each other against all the four test organisms, as revealed by One way ANOVA followed by Tukey’s t-test. Also, the individual effect of phytoconstituents and antibiotics on the four test organisms showed statistically significant difference (*p* ≤ 0.05)*.*

### Antimicrobial potential of the phytoconstituents against the resistant clinical isolates

The most active phytoconstituents, i.e., flavonoids and cardiac glycosides, when tested against the 34 drug resistant clinical isolates of *E. coli* and *Salmonella* spp. (CRIRS1–22) (Group 1) as well as MRSA (DSECI01–12) (Group 2) and an *Enterococcus* sp., showed a significant antimicrobial potential with overall Inhibition Zone (IZ) ranging from 12.33–28.33 mm (Table 4). Among the two phytoconstituents, flavonoids showed the highest and broad spectrum antimicrobial activity against the 33 out of total 34 clinical isolates, with IZ ranging from 14.66–28.33 mm, while Cardiac glycosides were active only against 14 out of total 34 clinical isolates with an IZ range of 12.33–17.33 mm. This difference in the antimicrobial efficacy of the two phytoconstituents was found to be statistically significant (*p* ≤ 0.05), as revealed by one way ANOVA followed by Post hoc Tukey’s t-test (Table 4).

**Table 4:** Antimicrobial potential of the most active phytoconstituents of *Symplocos racemosa* bark against drug- resistant strains and some clinical isolates of MRSA

	Microorganisms	Average zone of inhibition (mm)^*^	
		Flavonoids	Cardiac glycosides
	CRIRS 1	15.33 ± 0.881	–
	CRIRS 2	15.66 ± 0.333	–
	CRIRS 3	16 ± 0.577	–
	CRIRS 4	17.33 ± 0.333^a^	14.66 ± 0.333^b^
	CRIRS 5	17 ± 0.577 ^a^	12.33 ± 0.333^b^
	CRIRS 6	16.66 ± 0.333^a^	13 ± 0^b^
	CRIRS 7	22.66 ± 0.666	–
Group 1	CRIRS 8	17.66 ± 0.333^a^	13.66 ± 0.333^b^
	CRIRS 9	14.66 ± 0.881	–
	CRIRS 10	15.33 ± 0.881^a^	14.33 ± 0.333^a^
	CRIRS 11	15 ± 0.577	–
	CRIRS 12	15.66 ± 0.666	–
	CRIRS 13	19.66 ± 0.333^a^	14 ± 0^b^
	CRIRS 14	18.66 ± 0.333	–
	CRIRS 15	14.66 ± 0.333	–
	CRIRS 16	16.33 ± 0.881	–
	CRIRS 17	20 ± 0	–
	CRIRS 18	19.33 ± 0.333	–
	CRIRS 19	16 ± 0	–
	CRIRS 20	15.33 ± 0.333	–
	CRIRS 21	17.66 ± 0.333	–
	CRIRS 22	18.66 ± 0.333	–
	DSECI 01	21.33 ± 0.333^a^	14 ± 0^b^
	DSECI 02	20 ± 0^a^	17.33 ± 0.666^b^
	DSECI 03	27.66 ± 0.333^a^	16.66 ± 0.881^b^
	DSECI 04	28.33 ± 0.333^a^	18 ± 0 ^b^
Group 2	DSECI 05	24.33 ± 0.666^a^	15 ± 0 ^b^
	DSECI 06	22.33 ± 0.333^a^	14.33 ± 0.333^b^
	DSECI 07	26.33 ± 0.666	–
	DSECI 08	22 ± 0.577^a^	14.33 ± 0.666^b^
	DSECI 09	25.66 ± 0.333	–
	DSECI 10	27.33 ± 0.333	–
	DSECI 11	–	–
	DSECI 12	23.33 ± 0.333^a^	15.66 ± 0.333^b^

Similar alphabetic letters (a, a) on the values within a row represent no significant difference at 95% confidence level, as indicated by One-way ANOVA followed by Tukey’s t-test

^*^Values are expressed as Mean ± Standard error of means (SEM) (*n* = 3)

In case of Group 1 (CRIRS1–22)*,* i.e.*,* clinical isolates of *E. coli* and *Salmonella* spp., flavonoids showed a broad spectrum potential against all the 22 strains (14.66–22.66 mm) belonging to this group (Table 4), with an average Inhibition Zone (IZ) of 17.05 mm. Cardiac glycosides were active against only 6 organisms (CRIRS 4, 5, 6, 8, 10, 13) out of 22 strains, with an overall average IZ of 3.72 mm (Table 4). Hence, the flavonoids were more effective than the cardiac glycosides, since the difference in the average Inhibition Zone (IZ) of the two phytoconstituents against Group 1 (CRIRS1–22) isolates was found to be statistically significant (*p* ≤ 0.05), as revealed by one way ANOVA followed by Post hoc Tukey’s t-test.

Further, Flavonoids were significantly effective against 10 out of 11 strains of MRSA (DSECI 01–11) (Group 2), with IZ ranging from 20 to 28.33 mm, giving an average IZ of 22.30 mm. Here, DSECI 04 was the most sensitive (28.33 mm), while DSECI 11 was completely resistant. Cardiac glycosides were effective against 8 out of 12 strains, where the IZ ranged from 14 to 18 mm; hence giving an average IZ of 9.96 mm. DSECI04 was the most sensitive (18 mm), whereas the strains DSECI07, DSECI09, DSECI10 and DSECI11 were completely resistant. Hence, flavonoids showed a higher antimicrobial effectiveness than the cardiac glycosides, since the difference in the average Inhibition Zone (IZ) of the two phytoconstituents against DSECI 01–11) (Group 2) isolates was found to be statistically significant (p ≤ 0.05), as revealed by one way ANOVA followed by Post hoc Tukey’s t-test.

The *Enterococcus* sp. (DSECI 12) was susceptible to both flavonoids and cardiac glycosides, where flavonoids were more active (23.33 mm) than cardiac glycosides (15.66 mm) and this difference was statistically significant (p ≤ 0.05). Because of their effectiveness, the flavonoids were further tested against the 9 most susceptible strains (CRIRS7, CRIRS8, CRIRS13, CRIRS17, DSECI03, DSECI04, DSECI07, DSECI09 and DSECI10) for their MIC and MBC. The MIC values ranged from 15 to 35 mg/mL, lowest being against CRIRS7, DSECI07 and DSECI10 (15 mg/mL). The corresponding MBC values ranged from 20 to 40 mg/mL (Table T3 in Additional file 5).

### In vitro cytotoxicity studies by MTT assay against RD, L20B and Hep2 cell lines

The Flavonoids were further evaluated for their cytotoxicity against some cancerous cell lines, where a cytotoxic effect was seen with IC_50_ ranging from 361 to 494 μg/mL. The lowest IC_50_ (361 μg/mL) was seen against Hep2 cell line, whereas the highest IC_50_ was observed against RD cell line. IC_50_ for L20B cell line was 448 μg/mL (Fig. A4 in Additional file 5). In case of RD, complete rounding off of the cells was seen upto the concentration of 2.5 mg/mL, and thereafter the inhibitory effect gradually reduced down the concentration gradient. For Hep2 and L20B, rounding off of the cells could be seen upto 1.25 μg/mL and 0.625 μg/mL, respectively. The flavonoids showed a negligible effect on the normal cell line, i.e., Vero cell line (only 9.56–15.24%), in comparison to the % inhibition obtained against the three cancerous cell lines at the various tested concentrations (Fig. A4 in Additional file 5), thus exhibiting a very high IC_50_ value (> 10,000 μg/mL), in comparison to that obtained against cancerous cell lines (361–494 μg/mL).

### In-vivo acute oral toxicity studies

**Fig. 2:**
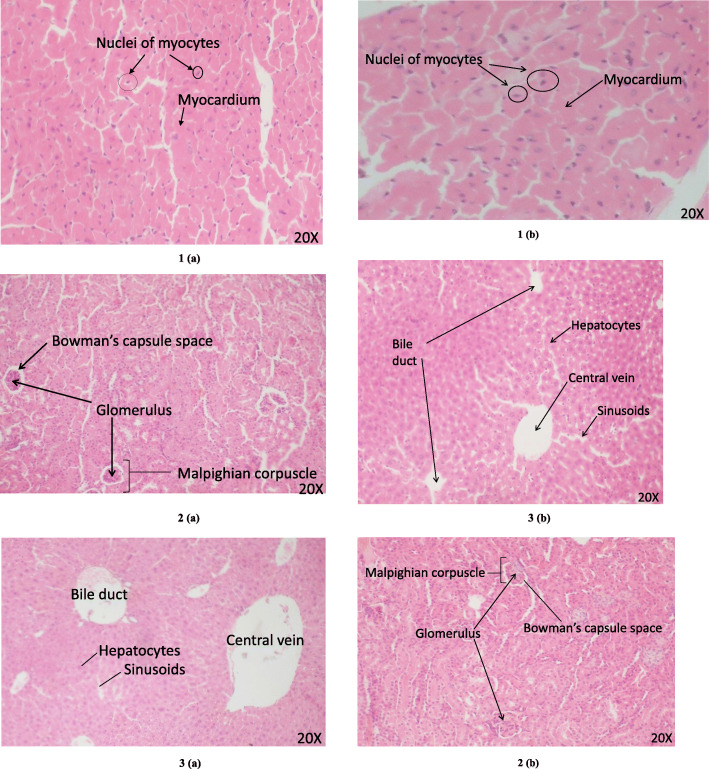
Photomicrographs of the tissue sections of (1) Heart (2) Kidney and (3) Liver taken from mice treated with *Symplocos racemosa* flavonoids (1**a**, 2**a** and 3**a**) and its comparison with their respective untreated control (1**b**, 2**b** and 3**b**)

The flavonoids, further tested for their toxicity in mice, did not show any toxic effects during the whole experimentation period as evident by the normal behavior and sleep patterns, salivation, breathing rate as well as the absence of any abnormalities on the skin, fur, eyes etc. No significant difference was seen between the weights of the tested (6/6) and the control (untreated) group (6/6) (Fig.A5 in Additional file 5). The weight of the vital organs obtained from each test group (6/6) showed no statistical difference from the control group (Table 5). The kidney and liver functioning remained normal in the test group (6/6) was completely normal since no statistical difference was seen between the various kidney and liver-functioning parameters of the treated and control groups of both male and female mice (Table 6). Further, the general and histopathological evaluation showed normal color, texture, architectural details and structural organization of the three organs under study (Fig. 2).

**Table 5:** Absolute and relative organ weight of control and treated mice (male and female) in the acute toxicity study of *Symplocos racemosa* flavonoids

	Absolute organ weight (g)^a^			Relative organ weight (%)^a^		
Organs	Control	Treated	*p*-value	Control	Treated	*p*-value
			**MALE**			
Liver	0.724 ± 0.031	0.692 ± 0.013	0.365	2.453 ± 0.031	2.441 ± 0.007	0.715
Kidneys	0.401 ± 0.022	0.379 ± 0.015	0.437	1.355 ± 0.035	1.334 ± 0.026	0.638
Heart	0.165 ± 0.007	0.157 ± 0.005	0.390	0.558 ± 0.008	0.577 ± 0.008	0.137
			**FEMALE**			
Liver	0.827 ± 0.044	0.783 ± 0.029	0.435	2.613 ± 0.055	2.653 ± 0.060	0.638
Kidneys	0.410 ± 0.023	0.368 ± 0.006	0.131	1.297 ± 0.031	1.248 ± 0.006	0.179
Heart	0.166 ± 0.0118	0.150 ± 0.004	0.254	0.524 ± 0.0195	0.509 ± 0.009	0.651

^a^Data indicate Mean ± SEM (*n* = 6 for each group). There was no significant difference between the test and control groups as indicated by Post hoc Tukey’s t-test

**Table 6:** Effect of *Symplocos racemosa* flavonoids on biochemical parameters in acute oral toxicity study

Parameters	Male			Female		
	Control^a^	Treated^a^	*p*-value	Control^a^	Treated^a^	*p*-value
Urea (Mmol/L)	42.633 ± 0.839	42.460 ± 0.553	0.867	40.13 ± 0.991	40.183 ± 0.539	0.963
Creatinine (μmol/L)	0.774 ± 0.026	0.765 ± 0.019	0.799	0.618 ± 0.024	0.601 ± 0.012	0.535
Total bilirubin (μmol/L)	2.376 ± 0.093	2.371 ± 0.063	0.882	1.76 ± 0.025	1.772 ± 0.014	0.677
Aspartate aminotransferase (AST)(U/L)	208.96 ± 4.192	209.11 ± 2.312	0.976	160.66 ± 2.708	159.76 ± 1.574	0.780
Alanine aminotransferase (ALT) (U/L)	79.95 ± 2.095	81.698 ± 0.591	0.441	61.33 ± 2.677	61.501 ± 1.338	0.955
Alkaline phosphatase (ALP) (U/L)	258.3 ± 2.510	256.89 ± 1.344	0.633	183.33 ± 1.612	181.733 ± 0.794	0.403

^a^Values are expressed as mean ± SEM (*n* = 6 for each group). There was no significant difference (*p* > 0.05) between the test and control groups as indicated by one way ANOVA followed by Post hoc Tukey’s t-test

### Detection of the probable bioactive components in *Symplocos racemosa* flavonoids

The bioactive compounds, which may be responsible for the antimicrobial efficacy of *Symplocos racemosa* flavonoids, were detected by separation using Preparative-Thin Layer Chromatography followed by their antimicrobial screening against two Gram positive, two Gram negative and one yeast strain. The most active band was then subjected to Gas Chromatography-Mass Spectrometry (GC-MS) to identify the antimicrobial components present in it.

#### Thin layer chromatography (TLC) analysis

**Fig. 3:**
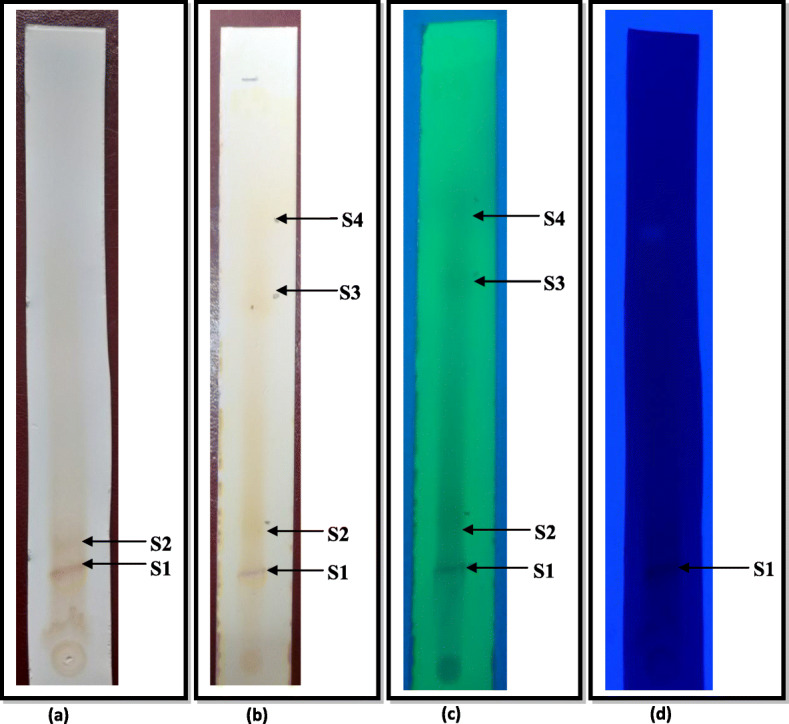
TLC chromatograms of the *Symplocos racemosa* flavonoids revealing the presence of various bands under **a** Natural light **b** Stained with Iodine vapors **c** UV light (264 nm) **d** UV light (365 nm)

The flavonoids were best resolved using the solvent system consisting of Butanol, Ethanol and water in the ratio 4:1:2. This solvent system separated the flavonoids into 4 bands having a Retention Factor (R_f_) in the range of 0.151–0.769. When observed under natural light, only 2 Bands were visible. Band 1 was seen as a dark brown spot while Band 2 was a light brown spot (Fig. 3a). All the four bands were visible when observed in iodine vapors and UV short wavelength (264 nm). Upon staining with iodine vapors, Band 1 was seen as the intensely brown spot and the other three bands showed light-brown intensity (Fig. 3b), whereas these bands could be seen as blue spots under UV short wavelength (264 nm). Here, Band 1 was intense in comparison to the other three bands (Fig. 3c). When observed under long wavelength UV at 365 nm, only Band 1 could be seen as a dark blue spot against the blue background while the other bands were not visible (Fig. 3d).

#### Quantitative separation of the bands using preparative-TLC (P-TLC) method and their antimicrobial screening

In case of *Symplocos racemosa*, 76.66 mg dry wt. of the flavonoids, when subjected to P-TLC, resulted in the separation of 4 bands (S1-S4) which were obtained with a dry wt. of: S1 (5 mg), S2 (8 mg), S3 (12 mg), S4 (9 mg). These were dissolved in methanol and a 20 μL aliquot was tested for their antimicrobial potential. Band S3 was significantly active against all the five test organisms. Band S1 was weakly effective against *Klebsiella pneumoniae* 1 and *Candida albicans* but was quite active against *Staphylococcus epidermidis.* Band S2 was negligibly active against *Staphylococcus aureus* and *Candida albicans*, but did not show any activity against *Klebsiella pneumoniae* 1, *Staphylococcus epidermidis* and *Shigella flexneri*. Band S4 was only active against *Staphylococcus epidermidis.* Since, Band S3 was significantly active against the test organisms; it was taken up further for spectroscopic studies.

#### Identification of the compounds present in the active band 3 (S3) by Gas Chromatography-Mass Spectrometry (GC-MS) analysis

**Fig. 4:**
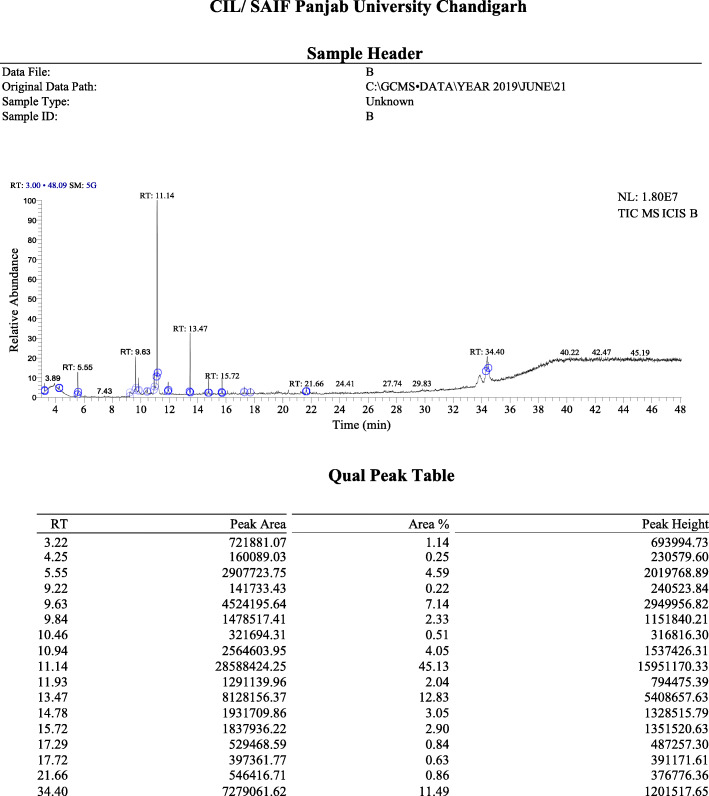
The GC-MS Chromatogram of the active band S3 obtained from the flavonoids of *Symplocos racemosa* bark

*Symplocos racemosa* flavonoids, when subjected to P-TLC analysis revealed the presence of 4 bands, out of which Band 3 (S3) was found to be antimicrobially most active, when screened against various test organisms. Its GC-MS analysis revealed the presence of mixture of a number of compounds at RTs falling within the range of 3.22–34.40 (Fig. 4; Additional file 6). The library search led to detection of a number of compounds at each RT (Table 7). Based on the abundance in terms of area %, compounds at three RTs were found to be major compounds in this active band, which were identified as Bicyclo [2.2.1]heptan-2-one,1,7,7-trimethyl-, (1S)- at an RT of 11.14; Silane, Dimethyl(dimethyl(dimethyl(2-isopropylphenoxy)silyloxy)silyloxy)(2-isopropylphenoxy)- at an RT of 13.47 and 7,11b-Dihydro-6H-indeno[2,1-c] chromene-3,4,6a,9,10-pentol pentakis (trimethylsilyl)ether at an RT of 34.40.

**Table 7:**
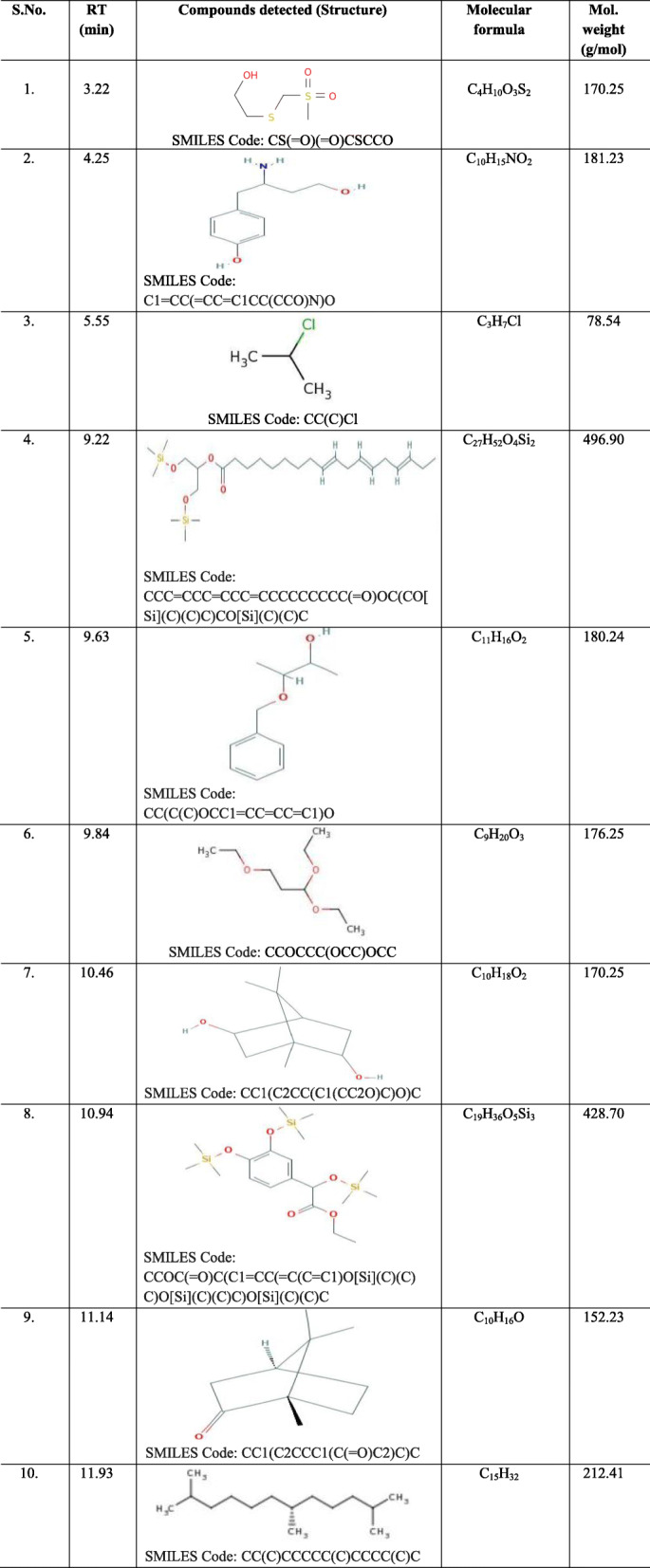

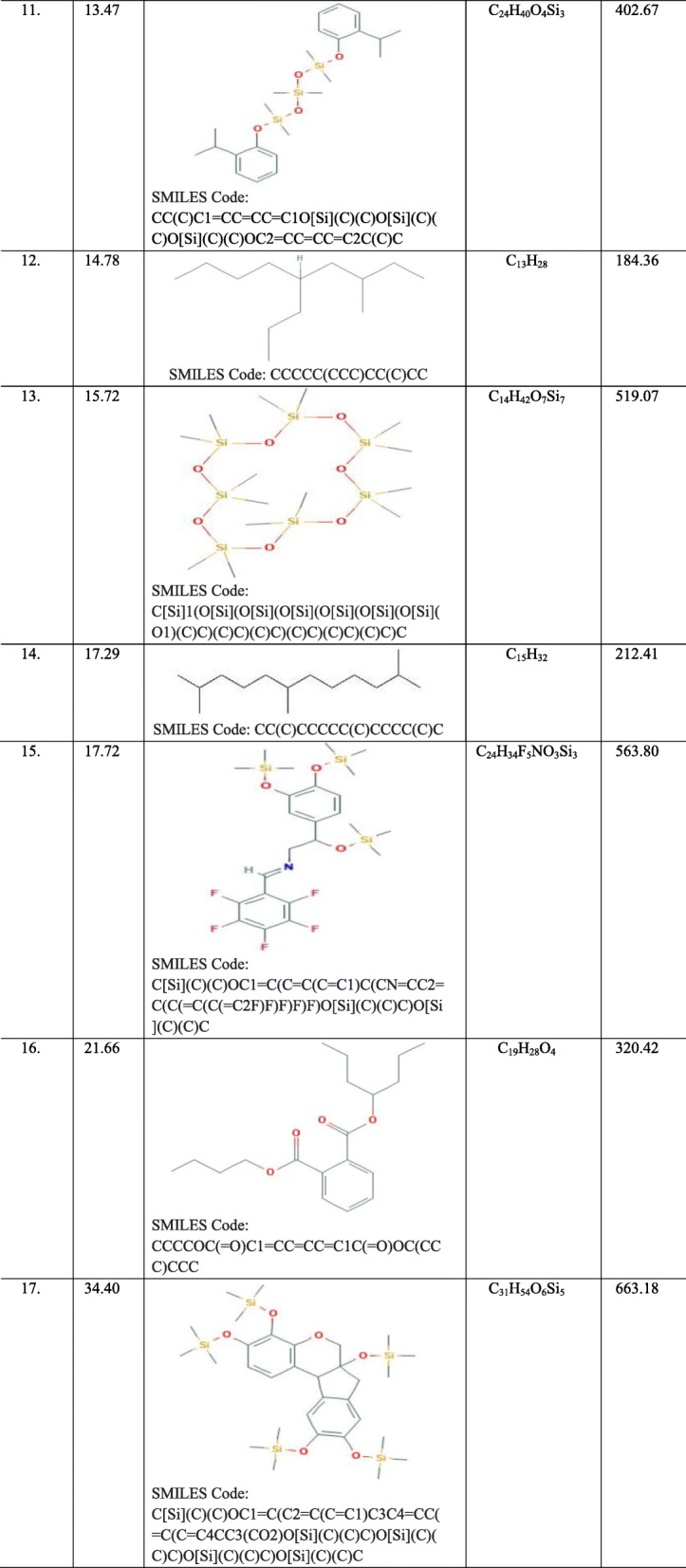
The compounds detected in the active band S3 obtained from the flavonoids of *Symplocos racemosa* bark using GC-MS

## Discussion

Antibiotics have always been bliss for the human civilization; however, their irrational and irresponsible use has significantly contributed to antimicrobial resistance. There is a promising future for the medicinal plants’ usage and their hidden potential could be decisive in changing the course of medical history [37]. Keeping this in mind, the present study aimed at scientifically exploring the bioactive potential of *Symplocos racemosa*. It has been observed that most of the antimicrobial compounds identified in plants are mostly aromatic or saturated organic molecules, which can easily be solubilized in organic solvents [38] and in the present study, ethyl acetate was able to extract out the antimicrobial components from *Symplocos racemosa* to a maximum extent, followed by butanol and hexane. The better antimicrobial potential of the ethyl acetate extract may be attributed to the better solubility of such residues in ethyl acetate [39]. The presence of major groups like flavonoids, cardiac glycosides, saponins, tannins, triterpenes and phytosterols are in consonance with other reports on this plant [40, 41] and other medicinal plants [17, 42]. Cardiac glycosides were the most abundant followed by flavonoids whereas triterpenes were present in least quantity. Quite encouragingly, flavonoids and cardiac glycosides were effective against 12 and 9 strains respectively and our results are well supported by the previous observations for various plant parts of *Moringa oleifera* [43, 44], where these were quite effective against the Gram positive, Gram negative and the yeast strains. The significance of the study can be gauged from the fact that these phytoconstituents were highly effective against resistant microbes e.g., MRSA and *Candida albicans*, which cause severe infections in hospitals/healthcare settings, which is in line with another study on *Eugenia jambolana* seed extracts which was effective against MDR human pathogens [45]. The antimicrobial potential of phytoconstituents is well-supported by the fact that these substances serve as plant defense mechanisms against predation by microorganisms and that the wide array of such microbial infections could have triggered the synthesis of such vast variety of phytoconstituents with a wide and variable spectrum [38]. The results obtained from ADA and MIC is supportive of each other. MIC determination is an effective method for evaluating the efficacy of an antimicrobial [46], where all the extracts were quite effective against MRSA, *Candida albicans, Pseudomonas aeruginosa* and *Escherichia coli* etc., which highlights the importance of the study. The MIC values obtained in this study (0.5–10 mg/mL) were comparable to or lower than the values obtained for other medicinal plants’ extracts, viz., 0.4–4 mg/mL [22]; 2.5–5 mg/mL [47], 5–20 mg/mL [48]. VCC studies give an assessment of the microbistatic/cidal effect of the compound, which further endorsed the potential of this medicinal plant, as it showed a significant bactericidal effect against MRSA, *Escherichia coli, Pseudomonas aeruginosa, Staphylococcus aureus, Staphylococcus epidermidis* etc. The PAE gives an idea of the time period upto which the compound is effective post-exposure and hence could be a decisive factor for development of any natural compound into a useful drug. The PAE in this study ranged from 2 to 6 h. The in vitro biosafety evaluation of the ethyl acetate extract and phytoconstituents by Ames and MTT assay proved them to be non-mutagenic and non-cytotoxic, respectively.

It is a well-known fact that the biofilms-formers are a leading cause of the rising cases of the chronic infectious diseases and recurrent infections. Therefore, bioactive compounds which are able to modulate/ disrupt the biofilm forming ability, have an exceptional importance in the field of drug development [49]. The promising antibiofilm potential of flavonoids and cardiac glycosides highlight the importance of the study, where the former were more effective [50]. This could be justified by the fact that the plant metabolites such as flavonoids, phenolic acids etc. reportedly possess antibiofilm activity [51, 52]. The potency was also tested against some drug-resistant strains of *E.coli, Salmonella* spp. and MRSA, where flavonoids and cardiac glycosides showed a broad spectrum activity, thus, justifying its candidature as a potential antimicrobial and antibiofilm drug of future. To add up on the bioactive potential of the plant, the most active phytoconstituents, i.e., flavonoids were screened for their cytotoxicity against three cancerous cell lines and our results are in consonance with those obtained for compounds of *Acanthus hirsutus* Boiss (11.17–700 μg/mL), *Euphorbia hirta* (625 μg/mL) [53, 54] and some naturally occurring quinones [55] .

To further strengthen the biosafety aspect, acute oral toxicity of its flavonoids was evaluated in mice. Toxicological evaluation is an important aspect of pharmacology for assessing any negative effect of the bioactive substance on living organisms prior to its clinical usage by humans. This aspect is crucially important as toxicity results from animals, especially mice and rats, help in judging the safety of the compounds, since mice and rats have an anatomy close to that of human body and any effect on the animal model would clearly indicate towards its possible toxic effects. Abundant literature is available validating the ethno-medical knowledge on the significance of medicinal plants in prevention and treatment of various diseases. However, the medicinal plants contain a complex mixture of many bioactive phytochemical groups with diverse mode of action, which may possibly show any adverse effects upon interaction with the human/animal cells. Thus, it is of utmost importance to investigate the safety and biological properties of the medicinal plants prior to their usage. The biosafety of the flavonoids was thus confirmed since no signs of abnormality, illness or biochemical and pathological changes were observed in the test group of animals, indicating a normal metabolism and growth. These results are in line with other allied studies reporting the non-toxicity of the medicinal plants/ extracts [12, 16–18].

So as to establish the active compounds which impart a significant and broad spectrum antimicrobial potential to the most active groups of phytoconstituents (flavonoids), the chromatographic and spectroscopic techniques such as TLC and GC-MS methods were employed. The chromatogram revealed the presence of several bands (S1-S4) with varying R_f_ values. The number of separated bands, corresponding to the number of separated compounds, strictly depended on the solvent system used. It means that the separation of bands depends on the nature and interaction of the compounds being separated with the solvents of the used system. The bands obtained were quantitatively separated using preparative-TLC method, where one band i.e.*,* S3 was found to be the active with a broad spectrum. Hence, it was further subjected to GC-MS analysis so as to identify the antimicrobial components in this active band. Interestingly, the presence of compound Bicyclo[2.2.1]heptan-2-one,1,7,7-trimethyl-, (1S)- as one of the major compounds in band S3 corresponded well with a report on the GC-MS analysis of the methanolic extract of *Coriandrum sativum* leaves, where this compound was identified as one of the major bioactive phytochemical compounds and possessed antimicrobial activity, which provided further credence to the study [56]. Its presence as a major phytochemical compound was also in concordance with other studies on chloroformic extract of *Acacia karoo* root and *Artemisia lavandulaefolia* essential oil, where Bicyclo[2.2.1]heptan-2-one,1,7,7-trimethyl-, (1S)- was present as a major bioactive phytochemical constituent [57, 58]. Also, another major compound “Silane, Dimethyl(dimethyl(dimethyl(2-isopropylphenoxy)silyloxy)silyloxy)(2-isopropylphenoxy)-” was also identified as a metabolic component of a wild edible mushroom *Pleurotus cornucopiae* (Paulet), by GC/MS [59]. Similarly, this compound has also been reported as a phyto-compound present in the extract of *Terminalia arjuna* upon its GC–MS analysis [60]. Similarly, compound “7,11b-Dihydro-6H-indeno[2,1-c] chromene 3,4,6a,9,10-pentol pentakis (trimethylsilyl)ether” has also been reported as a major bioactive components in the GC-MS analysis of the ethanol extract from leaves of *Combretum albidum* [61].

## Conclusion

The present study thus provides a scientific support to the use of *Symplocos racemosa* bark to explore its bioactive compounds of pharmaceutical importance, as evident from their wide zone of inhibition, low MIC values, shorter killing time and extended post antibiotic effect. These compounds showed a significant antibiofilm potential, were potent against drug resistant strains and were cytotoxic against the cancerous cell lines, which further add credence to the bioactive potential of the plant. The ethyl acetate extract and the phytoconstituents had a biosafe profile as seen in Ames test, MTT assay and acute oral toxicity studies. The various bioactivities exhibited by *Symplocos racemosa* revealed and strengthened its candidature for development of potent pharmaceutical drugs.

## Supplementary Information

**Additional file 1:.** Organic extracts’ preparation and their antimicrobial screening; Minimum Inhibitory Concentration (MIC); Qualitative and Quantitative analysis for the detection of major group of phytoconstituents; Ames Mutagenicity Test and MTT assay Protocol.

**Additional file 2: **Detailed protocol for Antibiofilm potential of the phytoconstituents of *Symplocos racemosa* bark; and Antimicrobial potential of the phytoconstituents against drug- resistant clinical isolates of MRSA, *Escherichia coli, Enterococcus* sp. and *Salmonella* spp.

**Additional file 3:.** In vitro cytotoxicity studies by MTT assay against RD, L20B and Hep2 cell lines.

**Additional file 4: **Acute Oral Toxicity study of *Symplocos racemosa* flavonoids in Swiss albino mice.

**Additional file 5: Figures A1-A6** and **Table TI, T2** and **T3** of the main manuscript.

**Additional file 6: **GC mass report of the compounds detected in band S3 of flavonoids from *Symplocos racemosa* bark.

## Data Availability

The raw datasets generated and/or analyzed during the current study are not publicly available [being a part of the Ph.D. thesis of the first author] but are available from the corresponding author on reasonable request.

## Abbreviations

ADA: Agar Diffusion Assay
CPCSEA: Committee for the Purpose of Control and Supervision of Experiments on Animals
IAEC: Institutional Animal Ethics Committee
MIC: Minimum Inhibitory Concentration
MRSA: Methicillin-Resistant *Staphylococcus aureus*
MTCC: Microbial Type Culture Collection
MTT: [3-(4,5-dimethylthiazol-2-yl)-2,5-diphenyl tetrazolium bromide]
PAE: Post Antibiotic Effect
TAP: Total Activity Potency
