# Supplementary material for: Bioprospecting the antimicrobial, antibiofilm and antiproliferative activity of Symplocos racemosa Roxb. Bark phytoconstituents along with their biosafety evaluation and detection of antimicrobial components by GC-MS
Source: BMC Pharmacol Toxicol. 2020 Nov 17;21:78. doi: 10.1186/s40360-020-00453-y (PMC7672880; doi:10.1186/s40360-020-00453-y)
Supplement: Supplementary file 1 — Additional file 1:. Organic extracts’ preparation and their antimicrobial screening; Minimum Inhibitory Concentration (MIC); Qualitative and Quantitative analysis for the detection of major group of phytoconstituents; Ames Mutagenicity Test and MTT assay Protocol. [file 40360_2020_453_MOESM1_ESM.docx]

**Organic extracts’ preparation and their antimicrobial screening**

Different organic solvents such as ethyl acetate, chloroform, butanol, hexane and methanol were screened to work out the best extractant. The organic extracts were prepared by using solvent-solvent extraction of the aqueous extract (detailed in Additional file 1). The aqueous extract was prepared by suspending the requisite amount of powdered plant material (17.5 g) in sterile distilled water (100 mL) and keeping it in hot water bath at 40°C for 20 min. Hundred milliliter of filtered aqueous extract was then shaken vigorously in a separating funnel with an equal volume of a particular organic solvent, for three independent times. The pooled organic layers were concentrated in a rotavapor at 45°C (under vacuum). The leftover material thus obtained were dissolved in diluted Dimethyl Sulfoxide [(30% (v/v)], which was then tested for their antimicrobial activity against the reference strains using the Agar Diffusion Assay (ADA).

To carry out the Agar Diffusion Assay (ADA), 0.1 ml of the activated test organism was inoculated onto the suitable agar medium plates with a sterile swab. Wells (8 mm diameter) were cut out in the medium using sterilized stainless steel borer. The organic extract (0.1 mL of concentration: Ethyl acetate extract: 30.5 mg/mL; Butanol extract: 35.42 mg/mL; Hexane extract: 27.98 mg/mL; Chloroform extract: 26.44 mg/mL) was added to each well and the plates were incubated in an upright position at 37°C (bacterial cultures) and 25°C (yeast strains) for 18–24 h. The antimicrobial activity could be observed as a zone of inhibition around the wells. Any organism with inhibition zone <12 mm was considered as resistant to the extract. Here, 30% DMSO served as a negative control and the experiment was performed in duplicate.

**Minimum Inhibitory Concentration (MIC)**

The test preparations (ethyl acetate extract and the active phytoconstituents) were tested for their MIC against the reference test organisms (sensitive to the respective extracts in ADA), as per protocol followed in Arora and Sood [21] by agar dilution method. Stock solutions [Ethyl acetate extract: 30.5 mg/mL; Flavonoids: 54.75 mg/mL; Cardiac glycosides: 59.5 mg/mL] were prepared and the MIC was determined using a range of concentrations [*i.e*., from 0.01-5 mg/mL (ethyl acetate extract) and from 0.01-10 mg/mL (phytoconstituents)] against the test organisms. Hundred microliters (100 µL) of the activated test organisms (adjusted to 0.5 McFarland standards) was swabbed onto the solidified plates and incubated at 37°C (bacterial cultures) and 25°C (yeast strains) for 18-24 h. The lowest concentration of the test preparations which inhibited the microbial growth were taken as their MIC. The values were compared with that of the standard antibiotics.

**PHYTOCHEMICAL ANALYSIS**

1. **Qualitative analysis for the detection of major group of phytoconstituents**

Qualitative analysis was carried out using the powdered plant material by standard chemical methods as protocols mentioned in (Arora and Sood, 2017; Arora and Onsare, 2014c; Ezeonu et al., 2016; Kaur and Arora, 2009) and are described below:

**Alkaloids**: They were detected using Wagner’s, Meyer’s and Hager’s reagents separately. The tests were scored positive on the basis of brown precipitates, yellow precipitates and turbidity respectively. **Flavonoids**: Occurrence of pink or magenta red coloration, magenta coloration, bulky white precipitate and dark green precipitate, respectively in Shinoda test, Zinc–hydrochloride reduction, lead acetate and ferric chloride tests was considered as a positive result. **Saponins**: Formation of froth upon vigorous shaking indicated a positive test. **Tannins**: Appearance of brownish–green/blue–black coloration and gelatinous/bulky white precipitation in ferric chloride test and lead acetate test respectively is a positive indication. **Cardiac glycosides**: These were detected using Keller Killiani test, where formation of reddish brown ring at the interface indicated their presence. **Terpenoids**: Presence of triterpenes was detected using Salkowski’s test, with Golden yellow coloration as positive indication. Diterpenes were detected using copper acetate test, where formation of emerald green color indicated positive. **Anthranol glycosides:** 0.2 g of plant powder was suspended in 8  ml of 1  M HCl and hydrolyzed for 2h. Treatment of 2 ml of the hydrolysate with 5% ferric chloride solution, then an equal volume of benzene, which was then separated and treated with 10% ammonium solution, determined a formation of rose pink in ammonical layer in a positive case. **Phytosterols**: Were detected using Libermann Burchard’s test and Salkowski test. **Coumarins** Their presence was assayed by adding 3 ml of 10% NaOH to 2 ml of the aqueous extract, where formation of yellow color indicated positive test.

1. **Quantitative isolation of the detected phytoconstituents**

The phytoconstituents which were qualitatively detected in the plant material were quantitatively isolated by standard methods as per protocols given in (Arora and Sood, 2017; Arora and Onsare, 2014c; Ezeonu et al., 2016; Kaur and Arora, 2009)and are described below:

**Flavonoids:** Two gram of powdered plant material was extracted with 40ml of 80% aqueous methanol under shaking conditions for 24h at 30°C. The filtrate, thus obtained, was evaporated on a rotary evaporator and the residual dry weight was determined as flavonoids. **Saponins**: Ten gram plant powder was extracted in 50ml of 20% aqueous ethanol for 30min under shaking condition, and was thereafter kept in a water bath for 4h at 55°C. The filtrate obtained was reduced to 20 ml volume in a water bath at 90 °C, followed by three times extraction with 20ml of diethyl ether. The aqueous portion was retained, pooled and extracted twice with 60ml butanol. The butanolic layer was pooled and washed two times with 10ml of 5% NaCl. The butanolic portion was then concentrated and dried to obtain a constant dry weight of saponins. **Cardiac glycosides:** Plant powder (2g) was extracted three times with 40 ml of methanol under shaking conditions at 30°C for 24hrs. The combined filtrates were evaporated to dryness and then defatted with petroleum ether for 24hrs. Following decantation, the dried residue was redissolved in 50% aqueous methanol and was extracted three times with chloroform. The filtrates were pooled and concentrated to obtain the dry weight as cardiac glycosides. **Diterpenes:** Two gram of the plant powder was extracted three times in 40ml of 50% ethanol at 30 °C for 24h. The collected filtrates were pooled and evaporated to dryness. The dried residue was weighed as diterpenes. **Tannins:** For isolation of tannins, 2g of plant powder was extracted five times with a mixture containing 10ml of 8% sodium carbonate (Na_2_CO_3_) and 20 ml distilled water in the ratio 1:15 (w/v). A 10ml volume of HCl and 20ml formaldehyde was added to the combined filtrate and kept under reflux for 30 min. The mixture was filtered in a pre weighed Whatmann paper**,** which was oven-dried to obtain a constant weight as tannins. **Phytosterols:** The isolation was carried out according to Samria and Sarin (2014) with slight modifications. Here, 2g of powdered plant material was defatted three times in petroleum ether for 24h on a water bath. The defatted material was air-dried and hydrolyzed with 50ml of 30% HCl (v/v) for 4h. The sample was repeatedly washed with distilled water till pH 7 was obtained. The sample was then dried and then extracted three times with 40ml of benzene for 24h. The filtrates were combined and dried *in vacuo* to obtain the dried mass as phytosterols

**Ames Mutagenicity Test Protocol**

The Ames test is a reversion mutation assay which employs a histidine auxotroph of *Salmonella* Typhimurium (MTCC 1251, IMTECH, Chandigarh) and was performed. The mutagenicity of the test extracts was determined on the basis of number of revertant colonies obtained upon exposure to the test compounds (due to any possible induced mutation) in comparison to a known mutagen (positive control). The assay was performed by the plate incorporation method, where the overnight activated culture was serially diluted upto 10^-3^ dilution. To the 5 ml of semi-solid top agar, were added 0.1 ml of the diluted inoculum, 0.1 ml of the test extract and 0.25 ml of the 0.5 mM histidine–biotin mixture (1:1 ratio) (0.5mM of histidine and biotin each were prepared separately, where the 0.5 mM biotin was dissolved in water at 60^◦^C to which the histidine solution was added). The contents were mixed thoroughly and immediately overlaid onto the solidified glucose minimal agar plates. The top agar was allowed to solidify and the plates were then incubated at 37°C for 24 h. The number of visible revertant colonies was counted. Sodium azide (5 μl of 17.2 mg/ml) acted as a positive control, while the respective diluents such as autoclaved distilled water and DMSO were used as negative control. The experiment was performed in duplicate and repeated thrice.

**MTT assay protocol**

In order to check the cellular toxicity of the extracts, MTT [3-(4, 5-dimethylthiazol-2-yl)-2, 5-diphenyl tetrazolium bromide] assay was performed. It is a colorimetric method based on the ability of mitochondrial succinate dehydrogenase enzyme of the metabolically viable cells to reduce the yellow-colored MTT dye into insoluble, purple-colored formazan crystals. In this assay, the sheep blood (10 ml) was mixed with the Alsever’s solution (anticoagulant) (3 ml) and transferred to sterile centrifuge tubes, which were centrifuged at 16,000 rpm for 15-20 min. The supernatant was discarded and the pellet obtained was washed thrice using 1 ml phosphate buffer saline (PBS). Upon washing, the pellet was finally resuspended in PBS. Various dilutions (10^-1^ to 10^-6^) of these blood cells were prepared using PBS. The cells in each dilution were counted with the help of a hemocytometer under light microscope and the dilution containing cells equivalent to 1×10^5^ cells/ ml was selected. Aliquots (100 μl) of the selected dilution were dispensed into a 96 well microtitre plate in duplicates, followed by overnight incubation at 37°C. After incubation, the supernatant was removed carefully from the wells and 200 μl of the test extract was added and incubated further for 24 h along with the control, where only the diluent (without compound) was added to the wells containing blood cells. Following incubation, the supernatant was removed and 20 μl MTT solution (5 mg/ml) was added to each well and incubated further for 3.5 h at 37°C under mild shaking (60 rpm). The dye was removed carefully and 50 μl DMSO was added to each well so as to dissolve the end product (formazan). The absorbance was then measured at 590 nm using microplate reader (Biorad 680-XR, Japan) and the % viability of the cells was thereby calculated.
