# Supplementary material for: Bioprospecting the antimicrobial, antibiofilm and antiproliferative activity of Symplocos racemosa Roxb. Bark phytoconstituents along with their biosafety evaluation and detection of antimicrobial components by GC-MS
Source: BMC Pharmacol Toxicol. 2020 Nov 17;21:78. doi: 10.1186/s40360-020-00453-y (PMC7672880; doi:10.1186/s40360-020-00453-y)
Supplement: Supplementary file 2 — Additional file 2: Detailed protocol for Antibiofilm potential of the phytoconstituents of Symplocos racemosa bark; and Antimicrobial potential of the phytoconstituents against drug- resistant clinical isolates of MRSA, Escherichia coli, Enterococcus sp. and Salmonella spp. [file 40360_2020_453_MOESM2_ESM.docx]

**Antibiofilm potential of the phytoconstituents of *Symplocos racemosa* bark**

The antibiofilm potential was tested against four organisms, *i.e*., *Escherichia coli*, *Staphylococcus aureus*, *Klebsiella pneumoniae* 1 and a yeast *Candida albicans* using protocol detailed in Additional File 2.

**Screening for biofilm formation**

The biofilm formation by test pathogens was screened out by the microtitre plate method as per protocol followed in Stepanovic *et al.* (2007) and Costa *et al.* (2018) with slight modifications. Tubes containing suitable broth (nutrient broth for bacteria; Yeast malt broth for yeast) were inoculated with a loopful of the test organism, which were then incubated at 37°C (bacteria) and 25°C (yeast) for 24h. The activated cultures were diluted upto 10^-2^ using respective broths and 200µl aliquot was dispensed into the 96-well microtitre plates in triplicates. The broth without the test microorganism acted as a negative control. The plate was then incubated at 37°C (bacteria) and 25°C (yeast) for 24h, following which the wells were decanted off and washed 3 times with phosphate buffer saline (pH 7.4) and then air dried followed by oven drying at 60°C for 60 min so as to heat-fix the remaining attached bacteria. Staining was then done with 0.1% crystal violet solution for 15min, followed by washing with deionized water and drying in inverted position. After the plate has air dried, the dye bound to the cells is eluted with 150µl of 95% ethanol by keeping at room temperature for approximately 30min without shaking. The 100 µl aliquot was transferred to a fresh microtitre plate and the intensity of biofilm formation was affirmed in terms of Optical density (OD) of stained adherent biofilm by using an ELISA reader (Biorad 680-XR, Japan) at 590 nm. The results are interpreted by firstly calculating the cut-off value (ODc), which separates biofilm producers from the non-biofilm producing strains, as follows:

Optical density cut-off value (ODc) = Average OD of the negative control + [3×standard deviation (SD) of negative control]. Based on the ODc values, strains may be divided into the following categories:

OD ≤ ODc (Non biofilm producer)

ODc < OD ≤ 2×ODc (weak biofilm producer)

2×ODc < OD ≤ 4×ODc (Moderate biofilm producer)

OD > 4×ODc (Strong biofilm producer)

Upon confirmation of the biofilm forming ability of the test organisms, the following assays were carried out to establish the antibiofilm potential of most active phytoconstituents.

**Inhibition of initial cell attachment**

The initial stage in biofilm formation is the attachment of planktonic cells on to a surface or a substrate. Therefore, exposure of the compounds with a promising antibiofilm potential may produce an unfavourable conditions for cellular attachment. The inhibitory potential of the partially purified phytoconstituents was carried out according to Jadhav *et al.* (2013) and Onsare and Arora (2015) with slight modifications. One hundred microlitres of the test compounds (flavonoid and cardiac glycosides) was added to the 96-well microtitre plates and an equal volume (100µl) of the cultures was then added to the wells to yield a final volume of 200 µL. Here, gentamicin and amphotericin B (1mgml^-1^) were added as standard compounds. The negative control consisted of inoculum without the test compounds. Sterile broth was added as an additional control to ensure that the media remained sterile during the course of the experiment. The plates were incubated at suitable temperature for 24h. Following incubation, the inhibition of initial cell attachment potential of the phytoconstituents in comparison with negative control (untreated cells) was established using crystal violet assay (described later). The antibiofilm potential of the phytoconstituents was further strengthened by checking their disruptive effect on the preformed biofilm, as described below.

**Screening of phytoconstituents for their disruptive potential of preformed biofilms**

It was performed as described earlier in Onsare and Arora (2015) and Costa *et al.* (2018) with slight modifications. One hundred microlitre (100µl) aliquot of each of the 4h activated cultures were dispensed into a 96-well microtitre plate and were incubated at 37°C/24h to allow biofilm formation. After incubation, the supernatant (broth) was discarded from all the sets and replaced with 100 µl fresh broth. An equivalent amount (100µl) of the test agents and standard antibiotics (1mg/ml) was added to the preformed biofilms, while in negative controls (untreated biofilms) the test extract/ antibiotic was substituted with sterile broth. The plates were further incubated at 37°C for bacteria and 25°C for yeast, and biomass content was estimated after 24h by the crystal violet assay.

**Estimation of biofilm biomass by Crystal Violet (CV) assay**

The estimation of biofilm biomass was assessed after the specific incubation periods for each of the above experiments by Crystal Violet (CV) assay (Djordjevic *et al*. 2002; Nikolic *et al.* 2014; Onsare and Arora 2015) with slight modifications. Crystal violet stains the cell membranes of attached cells, thereby giving an estimate of the cell density. Following the treatment of the test organisms, the culture medium from each well was discarded and plates were washed three times with sterile distilled water to remove any loosely adhered cells followed by air drying and then oven drying at 60°C for 45 min. The dried wells are then stained with 100 µl of 0.1% crystal violet and incubated at room temperature for 15 min. The plates were then repeatedly washed several times with sterile distilled water to remove unabsorbed stain. Absolute ethanol (125 µl) was added to destain the wells. One hundred microlitre of this solution was then transferred to a new plate and the absorbance determined at 590 nm to quantitatively estimate the biofilm density using an automated microplate reader (Bio-Rad 680-XR, Japan). The mean absorbance (OD_590_) of test was determined and the percentage inhibition was calculated using the formulae:

Percentage inhibition= 100- [{OD_590nm_ test well/ OD_590nm_ negative control well without antimicrobial agent} × 100].

**Estimation of metabolic activity by XTT assay**

Post exposure to the test extracts (flavonoids, cardiac glycosides and standard antibiotics), the metabolic activity of the treated biofilms was assessed using the modified {2, 3-bis [2-methyloxy-4-nitro-5-sulfophenyl]-2H-tetrazolium-5-carboxanilide} (XTT) reduction assay with slight modifications (Slama *et al*. 2012; Onsare and Arora 2015). After incubation of the biofilm with test extracts for 24 hrs, the culture medium was discarded from each well and washed three times with distilled water. Following the washings, 200µl of menadione–XTT working solution [XTT salt (1 mgml^-1^ dissolved in PBS, filter sterilized and stored at -70^0^C) was mixed with 2.5 µl and 20 µl of menadione (10mM of menadione dissolved in acetone, filter sterilized for bacteria and yeast respectively)] was added in respective well and further incubated in dark for 2h at the suitable temperature. Upon incubation, 100µl from each well was transferred to fresh wells and the quantification of color change (to an orange water soluble formazan derivative) was done using microtitre plate reader (Bio-Rad 680-XR, Tokyo, Japan) at 490 nm. The mean absorbance values of test wells were determined in comparison to that of negative control.

**Antimicrobial potential of the phytoconstituents against drug- resistant clinical isolates of MRSA, *Escherichia coli, Enterococcus* sp. and *Salmonella* spp.**

The most active phytoconstituents were tested for their antibacterial activity against the drug-resistant strains such as *Escherichia coli* (CRIRS 1-12) [Group 1], *Salmonella* spp. (CRIRS 13-22) [Group 2], MRSA (DSECI 01-11) [Group 3] and one strain of *Enterococcus* sp. (DSECI 12). The clinical isolates of MRSA and *Enterococcus* sp. were procured from Shri Guru Ram Das Medical College and Hospital, Amritsar, Punjab, India. The work with MDR *Escherichia coli* and *Salmonella* spp. was carried out at Central Research Institute, Kasauli (H.P.), India.

The phytoconstituents were tested for their antimicrobial efficacy by Agar Diffusion Assay (ADA), against twelve strains of MDR *Escherichia coli*, ten strains of MDR *Salmonella* spp., eleven strains of MRSA and one strain of *Enterococcus* sp. The cultures were activated by 4 h incubation in the suitable broth and their turbidity was standardized to 0.5 McFarland standards. An aliquot (100 μl) of these actively growing cultures was spread onto the suitable medium in triplicates and 8 mm wells were cut out. These wells were seeded with 100 μl of the phytoconstituents and thereafter plates were incubated at suitable temperature for 24 h. The activity was noted in terms of inhibition zone (in mm).

**Determination of MIC and MBC values of phytoconstituents**

The Minimum Inhibitory Concentration (MIC) and Minimum Bactericidal Concentration (MBC) for the most active phytoconstituents was worked out against the most sensitive organisms by broth microdilution method in a 96-well microtitre plate in triplicate sets. The 2-fold serial dilutions of the phytoconstituents were prepared in the Mueller Hinton broth (MHB), with concentrations in the range of 0.09 mg/ml- 50 mg/ml. The 50 µl of the actively growing bacterial suspension (adjusted according to 0.5 McFarland standards) was added to each well, making up a total volume of 200 µl in each well. The inoculums without the test compound served as a negative control and the nutrient broth alone acted as a sterility control. The plate was incubated at 37°C for 24 h. The lowest concentration with no visible growth was defined as the Minimum Inhibitory Concentration (MIC). To determine the MBC, the aliquots from the wells showing no visible growth were swabbed onto the nutrient agar plates and incubated at 37°C for 24 h. The concentration corresponding with no growth on the plates was taken as MBC
