# Supplementary material for: Bioprospecting the antimicrobial, antibiofilm and antiproliferative activity of Symplocos racemosa Roxb. Bark phytoconstituents along with their biosafety evaluation and detection of antimicrobial components by GC-MS
Source: BMC Pharmacol Toxicol. 2020 Nov 17;21:78. doi: 10.1186/s40360-020-00453-y (PMC7672880; doi:10.1186/s40360-020-00453-y)
Supplement: Supplementary file 3 — Additional file 3:. In vitro cytotoxicity studies by MTT assay against RD, L20B and Hep2 cell lines. [file 40360_2020_453_MOESM3_ESM.docx]

***In vitro* cytotoxicity against RD, L20B and Hep2 cell lines by MTT assay**

The cytotoxic effect of the most active phytoconstituent (flavonoids) of *Symplocos racemosa* bark was studied against three cell lines (RD, L20B and Hep 2) by MTT [3-(4,5-dimethylthiazol-2-yl)-2,5-diphenyl tetrazolium bromide] assay as described previously (AL-Asady et al., 2014; Das et al., 2015; Harput et al., 2011) with slight modifications. The experiment was performed in triplicates.

**Cell lines and culture medium**

RD (Human Rhabdomyosarcoma), L20B (Diploid mouse lung cell line) and Hep 2 (Human epithelioma of larynx) cell lines were obtained from Central Research Institute (C.R.I), Kasauli, Himachal Pradesh, India. Stock cultures were prepared in a 25cm^2^ tissue culture bottle containing Dulbecco’s Modified Eagle’s Medium (DMEM) supplemented with 10% inactivated Fetal Bovine Serum (FBS), penicillin (100 IU/ml), streptomycin (100 µg/ml) and amphotericin B (5 µg/ml) at 37 °C in a humidified atmosphere (90% RH) and 5% CO_2_ level.

**Standardization of stock cultures**

The cell line monolayer in each 25cm^2^ bottle was trypsinized using 2 ml Trypsin Phosphate Versene Glucose (TPVG) solution (0.2% trypsin, 0.05% glucose and 0.02% EDTA in PBS). The TPVG solution was then carefully discarded and a cell suspension was prepared using 2ml of DMEM with 10% FBS. The cell count in this suspension was adjusted to 1x10^5^ cells/ ml with DMEM (containing 10% FBS) by trypan blue dye exclusion technique using Neubauer chamber.

**Preparation of test dilutions**

For the cytotoxicity studies, a weighed quantity of *Symplocos racemosa* flavonoids was dissolved separately in a known volume of 30% DMSO. A stock solution (10mg/ml) was prepared from it using DMEM supplemented with 2% inactivated FBS and sterilized by filtration (0.2 µm syringe filter). Two fold serial dilutions ranging from 10mg/ml to 0.039mg/ml were prepared from this stock solution and were used in the experimentation.

**Determination of cytotoxicity by MTT assay**

Hundred microlitre (100 µl) of the diluted cell suspension (approx. 10,000 cells) of each cell line was added to a separate 96 well microtitre plate and incubated in a CO_2_ incubator at 37°C containing 5% CO_2_ for 24h. After 24hrs, the supernatant was removed and the monolayer was gently washed with the medium containing 10% FBS. Different test concentrations of diterpenes (100µl) were then aseptically added to each of the microtitre plate containing cell line, where untreated cells and the diluent were taken as a untreated control. The plates were then incubated at 37°C with 5% CO_2_ atmosphere for 72h. The plates were checked every 24h for any signs of contamination. After 72h, the test dilutions were flicked off from each plate and 50 µl of the MTT solution (5mg/ml in PBS) was added to each well. All the microtitre plates were gently shaken and incubated at 37°C in a CO_2_ incubator for 3h. The solution was then carefully removed and 100 µl DMSO was added to each well so as to solubilize the formazan crystals. The absorbance was measured using a microplate reader at 540 nm. The percent growth inhibition was calculated using formula:

Mean OD of each test concentration

Mean OD of the positive control

% growth inhibition = 100 – x100

The concentration of the test compound (diterpenes) needed to inhibit the cell growth by 50% (IC_50_) was calculated from the dose-response curve generated for each cell line.
