# Supplementary material for: Bioprospecting the antimicrobial, antibiofilm and antiproliferative activity of Symplocos racemosa Roxb. Bark phytoconstituents along with their biosafety evaluation and detection of antimicrobial components by GC-MS
Source: BMC Pharmacol Toxicol. 2020 Nov 17;21:78. doi: 10.1186/s40360-020-00453-y (PMC7672880; doi:10.1186/s40360-020-00453-y)
Supplement: Supplementary file 5 — Additional file 5: Figures A1-A6 and Table TI, T2 and T3 of the main manuscript. [file 40360_2020_453_MOESM5_ESM.docx]

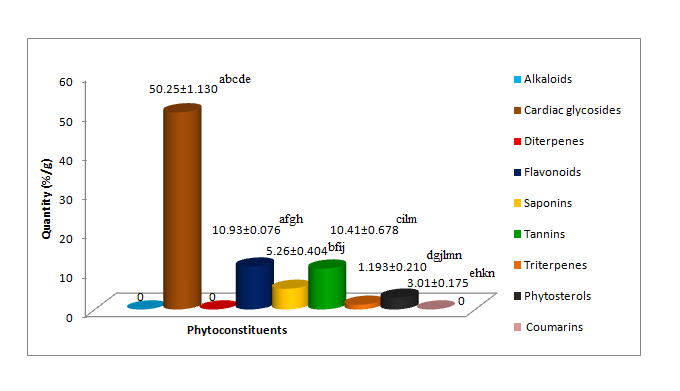
**Fig.A1:** The concentrations of various phytoconstituents of *Symplocos racemosa* bark (%/g). *The values are expressed as Mean ± SEM for N=3. **Same superscript alphabetic letters within the rows show significant statistical difference (p≤ 0.05) among test organisms as indicated by Post hoc Tukey’s t-test.


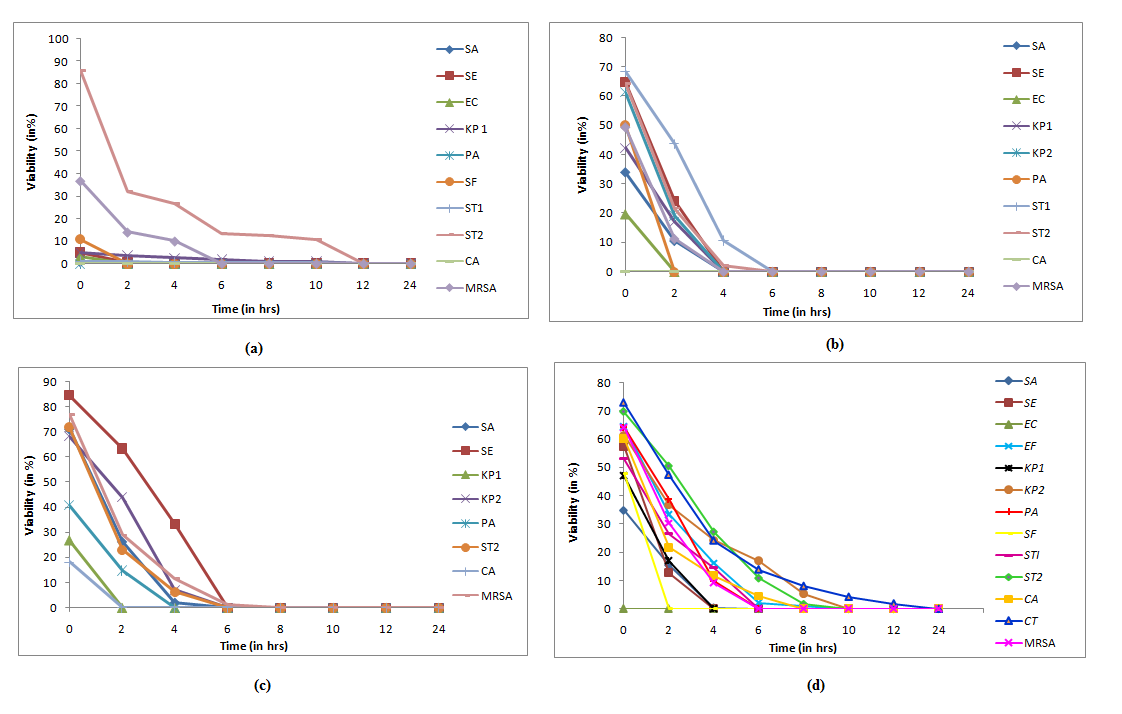


**Fig.A2**: Viable Cell Count studies of the (a) Organic extract (b) Flavonoids (c) Cardiac glycosides of *Symplocos racemosa* bark and (d) Gentamicin (*Amphotericin B for yeast strains). * **SA**- *Staphylococcus aureus*; **SE-** *Staphylococcus epidermidis*; **EC**- *Escherichia coli*; **KP1**- *Klebsiella pneumoniae* 1; **KP2**- *Klebsiella pneumoniae* 2; **SF**- *Shigella flexneri*; **ST1**- *Salmonella typhimurium* 1; **ST2**- *Salmonella typhimurium* 2; **PA**- *Pseudomonas aeruginosa*; **CA**- *Candida albicans*; **CT**- *Candida tropicalis*; **MRSA**- Methicillin- Resistant *Staphylococcus aureus.*


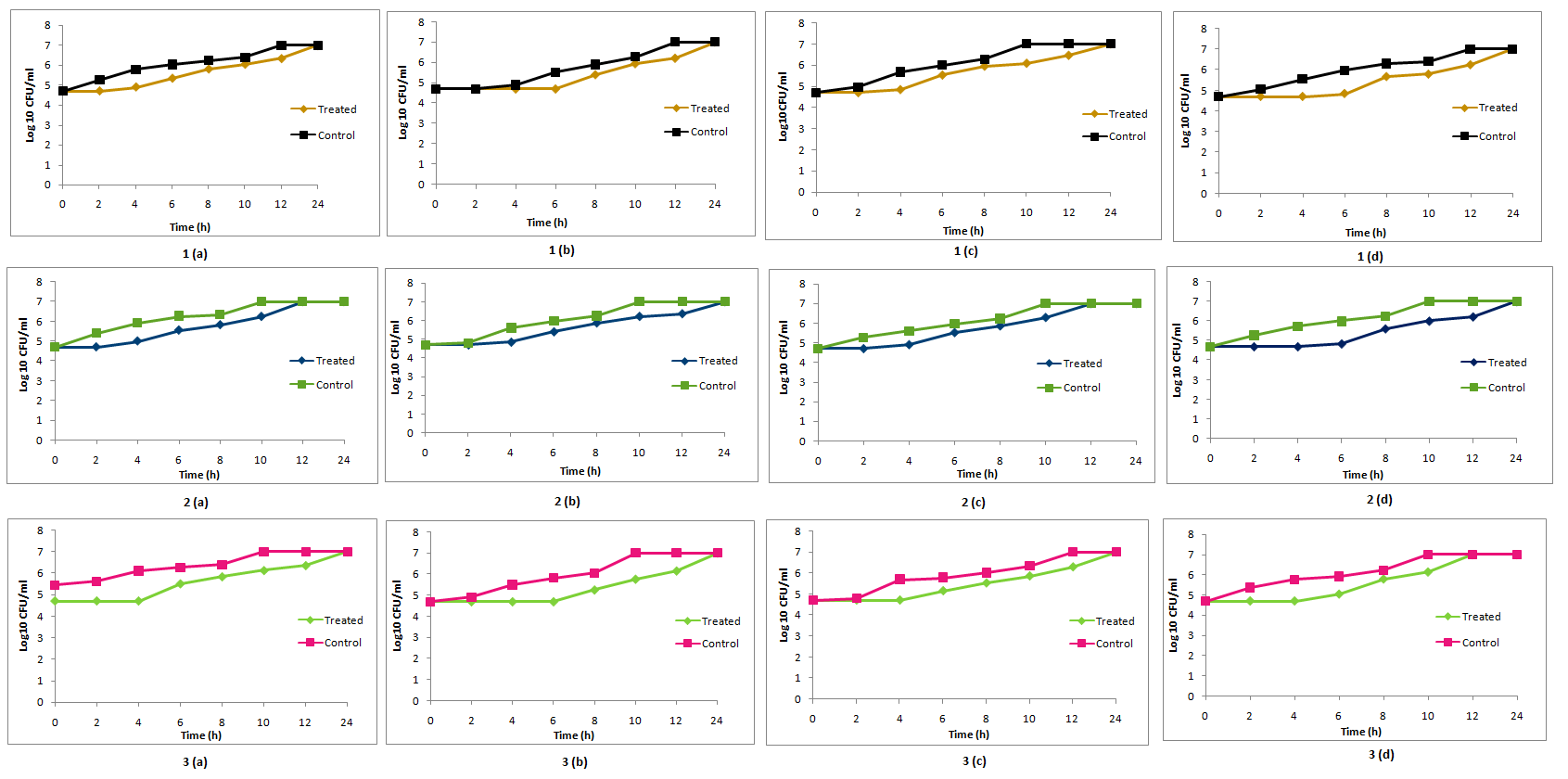


**Fig. A3:** Post Antibiotic Effect (PAE) of *Symplocos racemosa* bark of (1) Ethyl acetate extract (2) Cardiac glycosides (3) Flavonoids against (a) *Staphylococcus aureus* (b) *Salmonella typhimurium* 2 (c) *Klebsiella pneumoniae* 2 (d) *Candida albicans*.

**(b)**

**(b))**

**(a)**

**(c)**

**(d))**

**(d)**

**Fig. A4:** *In vitro* cytotoxicity of *Symplocos racemosa* flavonoids on **(a)** L20B **(b)** RD **(c)** Hep2 **(d)** Vero cell line by MTT assay

**
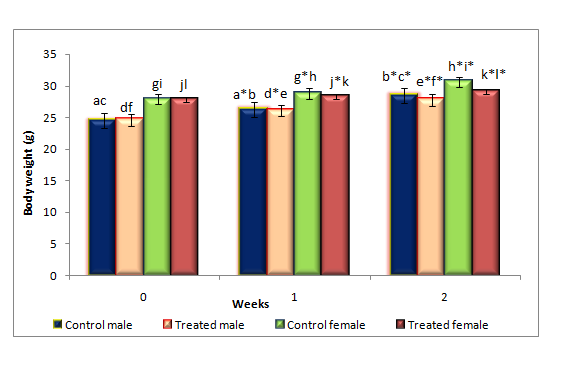
**

**Fig.A5:** Mean body weight of mice upon dosage of *Symplocos racemosa* flavonoids. Values are expressed as mean ± SEM (n=6 for each group). Same superscript alphabetic letters above the bars show significant statistical difference. Asterisk denotes (p≤ 0.05) as indicated by Post hoc Tukey’s t-test.


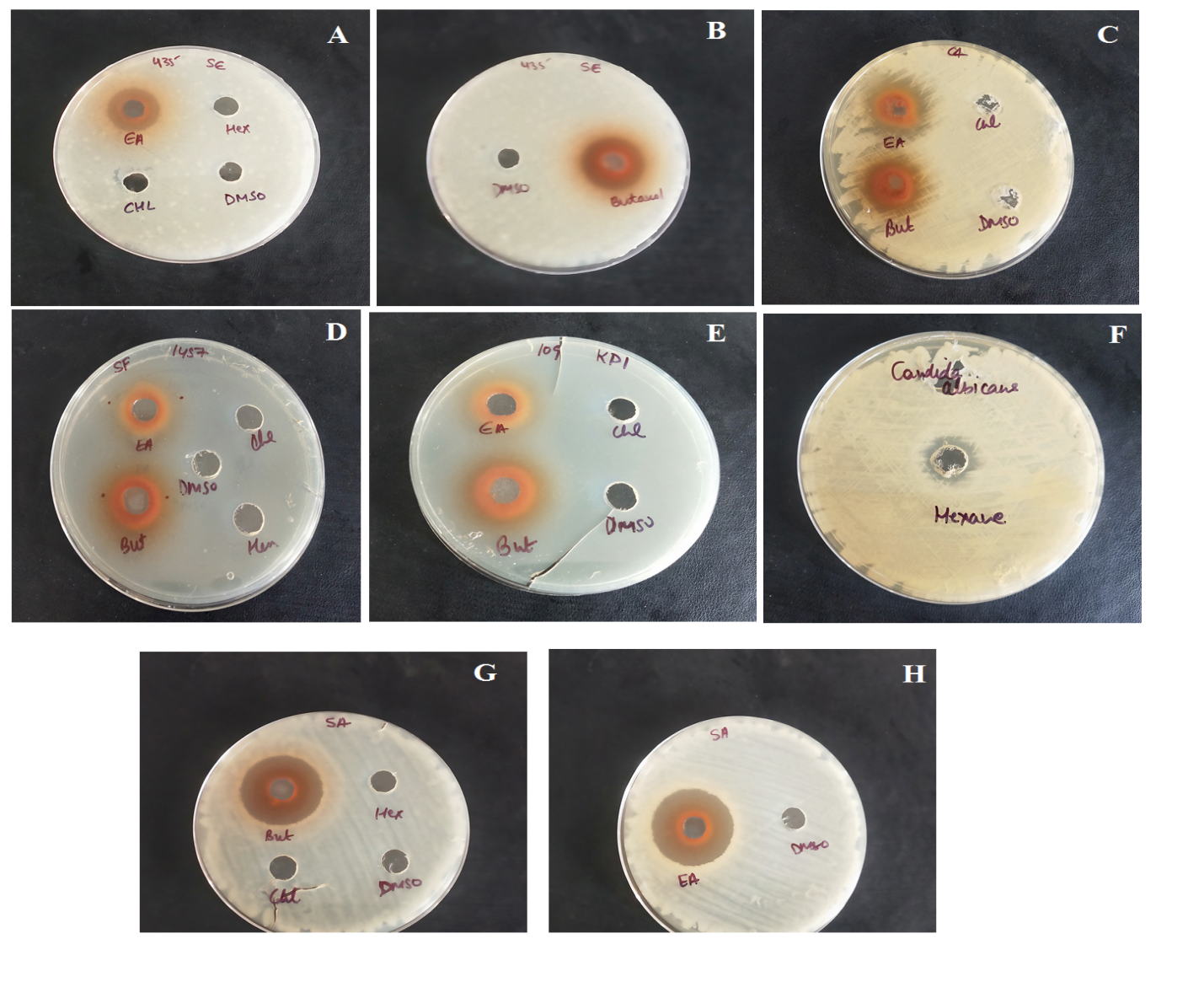


**Fig.A6:** Plates showing the antimicrobial activity of various solvent extracts of *Symplocos racemosa* against some representative microorganisms. **A-B**: *Staphylococcus epidermidis*; **C, F**: *Candida albicans*; **D*:*** *Shigella flexneri*; **E:** *Klebsiella pneumoniae* 1; **G-H:** *Staphylococcus aureus.*

**Table T1:** The antibiotic resistance profile of the drug resistant clinical isolates obtained from Central Research Institute, Kasauli (H.P.), India and Shri Guru Ram Das Medical College and Hospital, Amritsar, Punjab, India.

| **Organisms** | **Resistant strain** | **Resistant against antibiotics (µg/disc)*** |
| --- | --- | --- |
| *Escherichia coli* | CRIRS 1 | AMC (30), COT (25), CTX (30), NA (30), TR (5) |
|  | CRIRS 2 | NA (30), PIT (100/10), AMC (30) |
|  | CRIRS 3 | NA (30) |
|  | CRIRS 4 | AMC (30), AMP (10), COT (25), CTR (30), CTX (30), CXM (30), NA (30), C (30) |
|  | CRIRS 5 | AMC (30), AMP (10), COT (25), CTR (30), CTX (30), CXM (30), NA (30), TR (5) |
|  | CRIRS 6 | AMC (30), AMP (10), COT (25), CTR (30), CTX (30), CXM (30), NA (30), TR (5), NX (10) |
|  | CRIRS 7 | AMC (30), AMP (10), CPM (30), CTR (30), CTX (30), CXM (30), NA (30), TR (5), NX (10) |
|  | CRIRS 8 | AMC (30), AMP (10), COT (25), CTX (30), NA (30), TR (5) |
|  | CRIRS 9 | AMC (30), AMP (10), COT (25), CTR (30), CTX (30), CXM (30), NA (30), TR (5) |
|  | CRIRS 10 | AMC (30), AMP (10), COT (25), CTR (30), CTX (30), CXM (30), NA (30), TR (5) |
|  | CRIRS 11 | AMC (30), AMP (10), COT (25), NA (30), TR (5) |
|  | CRIRS 12 | NA (30), AMC (30) |
| *Salmonella* spp. | CRIRS 13 | AMC (30), AMP (10), CTX (30), NA (30), PIT (100/10) |
|  | CRIRS 14 | AMC (30), AMP (10), CTR (30), CTX (30), CXM (30), PIT (100/10) |
|  | CRIRS 15 | AMC (30), AMP (10), CTX (30), NA (30), PIT (100/10) |
|  | CRIRS 16 | CTX (30), NA (30), PIT (100/10), AMC (30), AMP (10) |
|  | CRIRS 17 | AMC (30), CTX (30), CXM (30), PIT (100/10) |
|  | CRIRS 18 | AMC (30), AMP (10), COT (25), CTX (30), NA (30), TR (5), PIT (100/10) |
|  | CRIRS 19 | COT (25), NA (30) |
|  | CRIRS 20 | AMC (30), AMP (10), CTX (30), CXM (30), GEN (10), NA (30), PIT (100/10) |
|  | CRIRS 21 | AMC (30), AMP (10), CTR (30), CTX (30), CXM (30) |
|  | CRIRS 22 | AMC (30), AMP (10), CIP (5), CTR (30), CTX (30), CXM (30), GEN (10), NA (30), NX (10), PIT (100/10) |
| *Enterococcus* sp. | DSECI 12 | CX (30), AMC (30), AZM (15) |
| MRSA** | DSECI 1-11 | MET (10), AK (30), GEN (10) |

***AMC:** Amoxyclav; **COT:** Co-trimoxazole; **CTX:** Cefotaxime: **NA:** Nalidixic acid, **TR:** Trimethoprim; **AMP:** Ampicillin; **CTR:** Ceftriaxone; **CXM:** Cefuroxime; **C:** Chloramphenicol; **NX**: Norfloxacin; **CPM:** Cefepime; **PIT**: Piperacillin/ Tazobactam**; GEN**: Gentamicin; **CX:** Cefoxitin; **AZM**: Azithromycin; **MET**: Methicillin; **AK**: Amikacin; ** Methicillin Resistant *Staphylococcus aureus*

**Table T2: Ames mutagenicity test and MTT assay of ethyl acetate extract and the phytoconstituents of *Symplocos racemosa***

1. **Ames Test**

| **Plant** | **Test compound** | **No. of revertant colonies** |
| --- | --- | --- |
| ***Symplocos racemosa*** | **Ethyl acetate extract** | **No colonies** |
|  | **Flavonoids** | **No colonies** |
|  | **Cardiac glycosides** | **No colonies** |
| **Positive control** | **Sodium azide** | **856** |

1. **MTT assay**

| **Plant** | **Test compound** | **Absorbance at 590 nm (OD_590_)** | | | **% Viability** |
| --- | --- | --- | --- | --- | --- |
|  |  | **Test** | | **Untreated Control** |  |
| ***Symplocos racemosa*** | **Ethyl acetate extract** | **0.411** | **0.428** | | **96.02%** |
|  | **Flavonoids** | **0.562** | **0.612** | | **91.83%** |
|  | **Cardiac glycosides** | **0.591** | **0.612** | | **96.56%** |

**Table T3**: **MIC and MBC values of the *Symplocos racemosa* flavonoids against some of the drug resistant strains**

| **Resistant strains** | **MIC (mg/ml)** | **MBC (mg/ml)** |
| --- | --- | --- |
| CRIRS7 | 15 | 20 |
| CRIRS8 | 30 | 35 |
| CRIRS13 | 20 | 25 |
| CRIRS17 | 25 | 30 |
| DSECI03 | 35 | 40 |
| DSECI04 | 35 | 40 |
| DSECI07 | 15 | 20 |
| DSECI09 | 25 | 30 |
| DSECI10 | 15 | 20 |
