# Supplementary figures and images for: Bioprospecting the antimicrobial, antibiofilm and antiproliferative activity of Symplocos racemosa Roxb. Bark phytoconstituents along with their biosafety evaluation and detection of antimicrobial components by GC-MS
Source: BMC Pharmacol Toxicol. 2020 Nov 17;21:78. doi: 10.1186/s40360-020-00453-y (PMC7672880; doi:10.1186/s40360-020-00453-y)

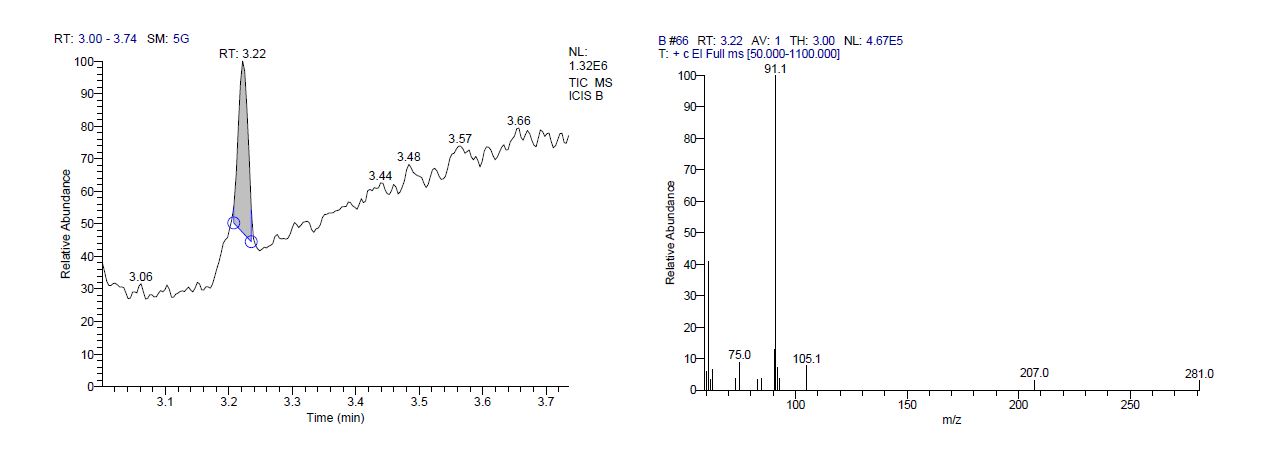


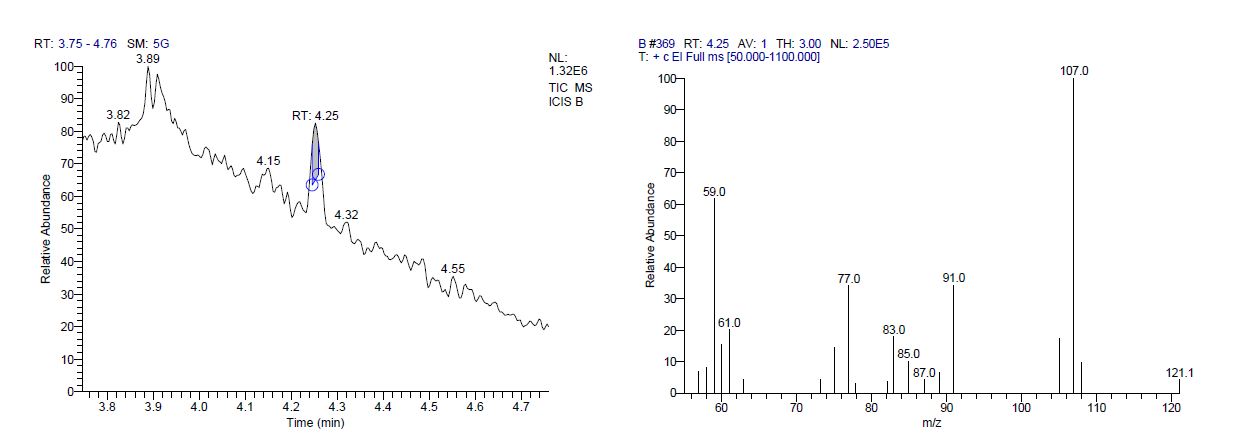


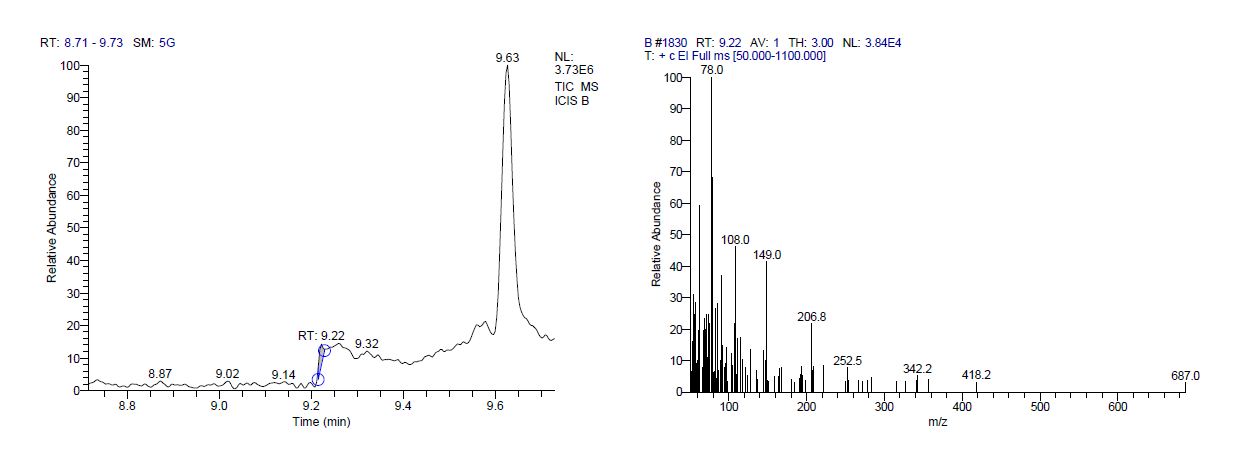

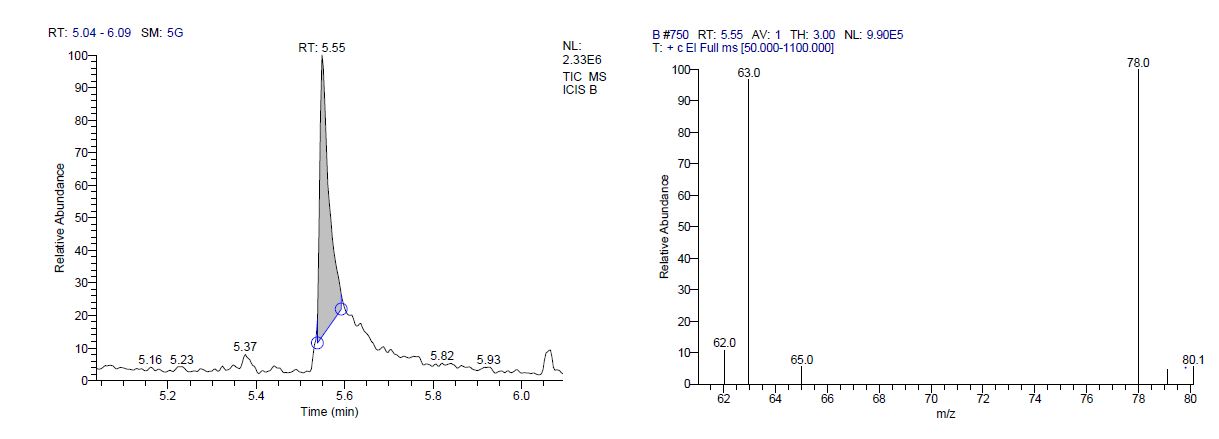


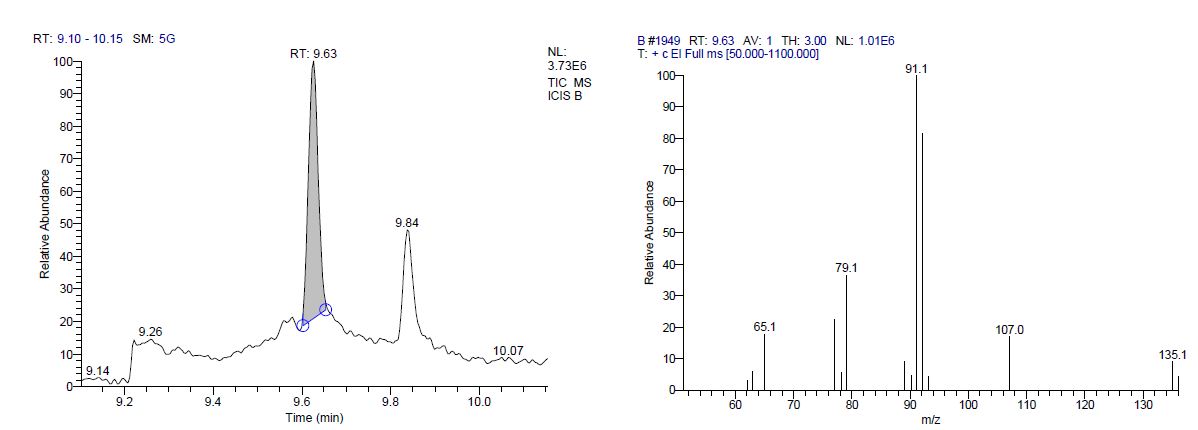


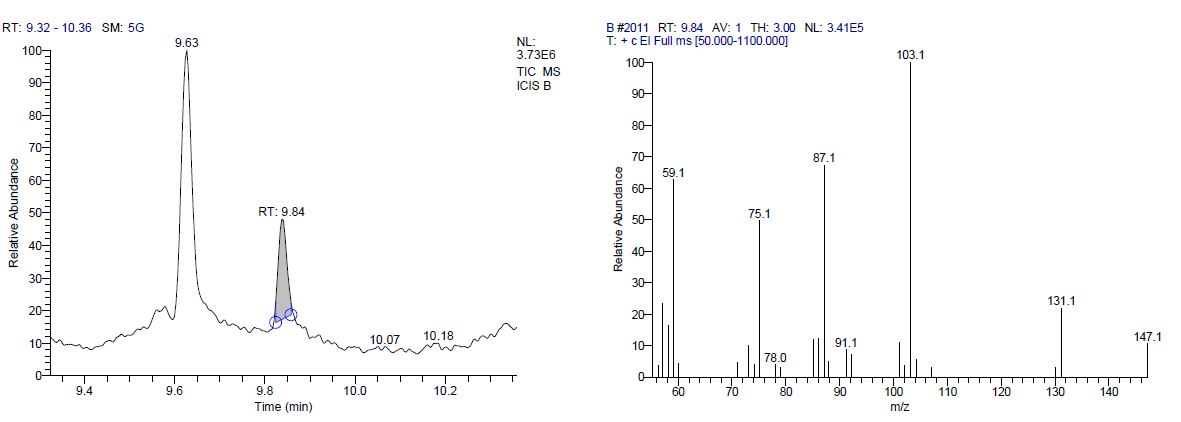


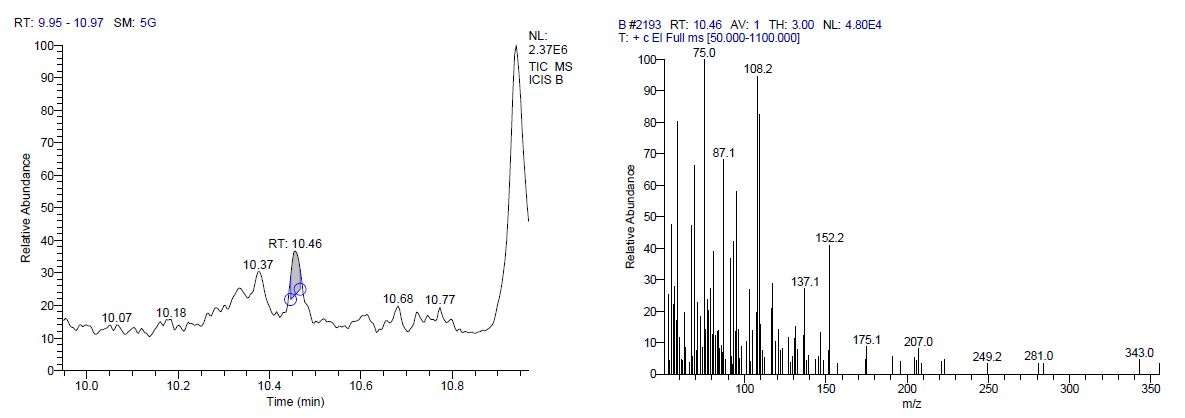


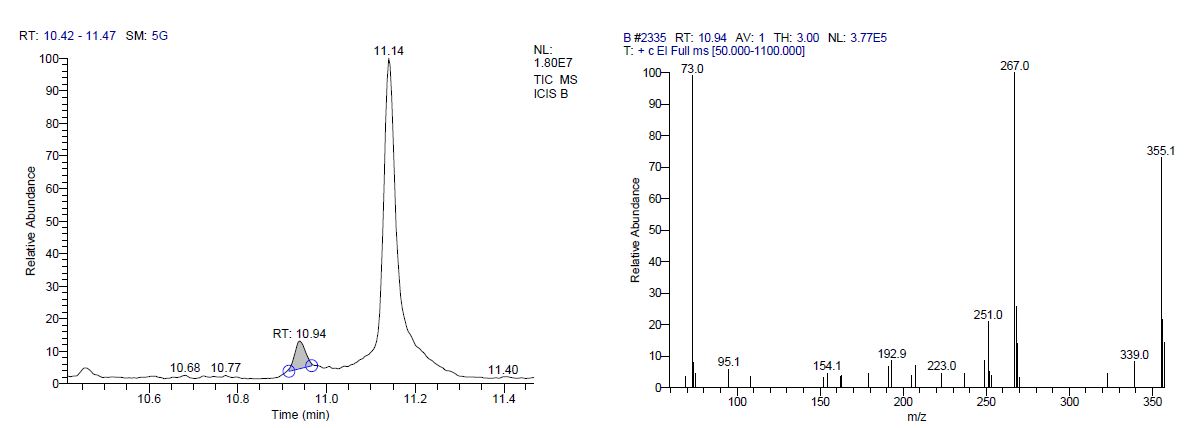


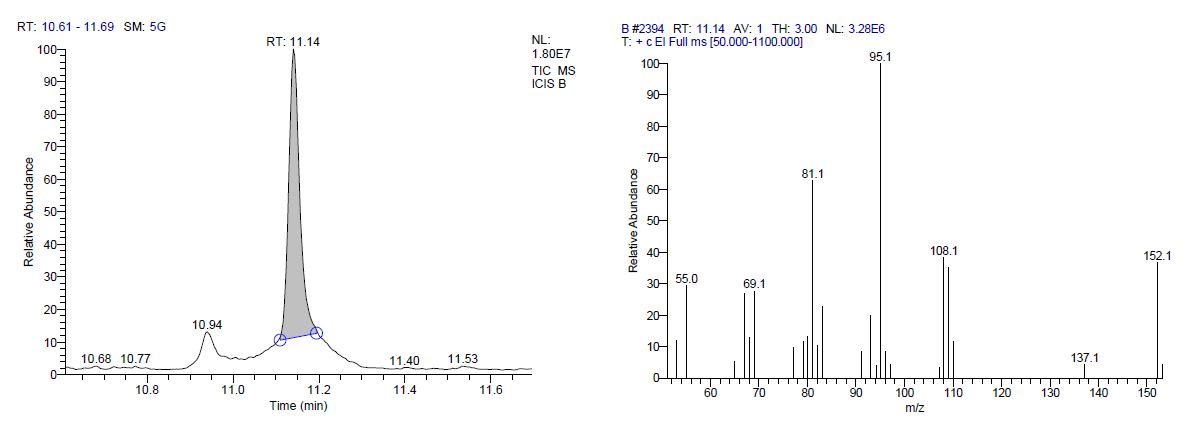


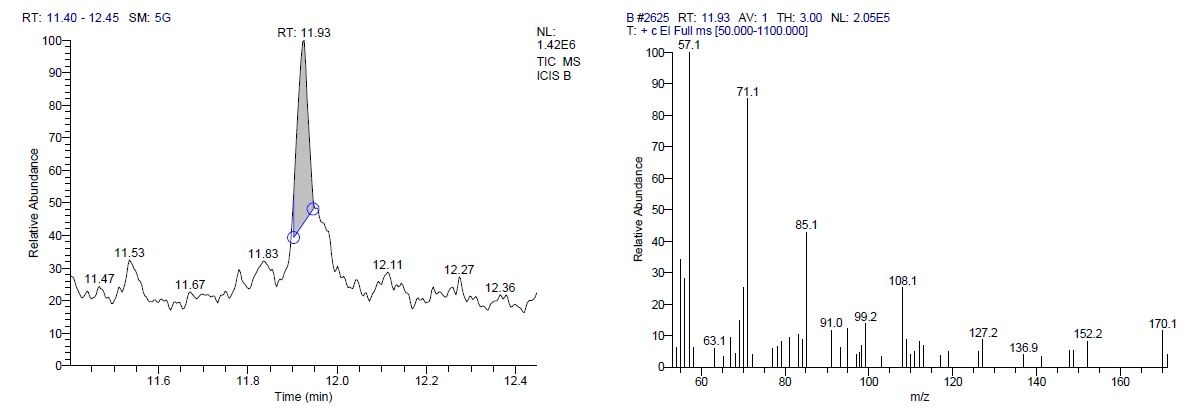


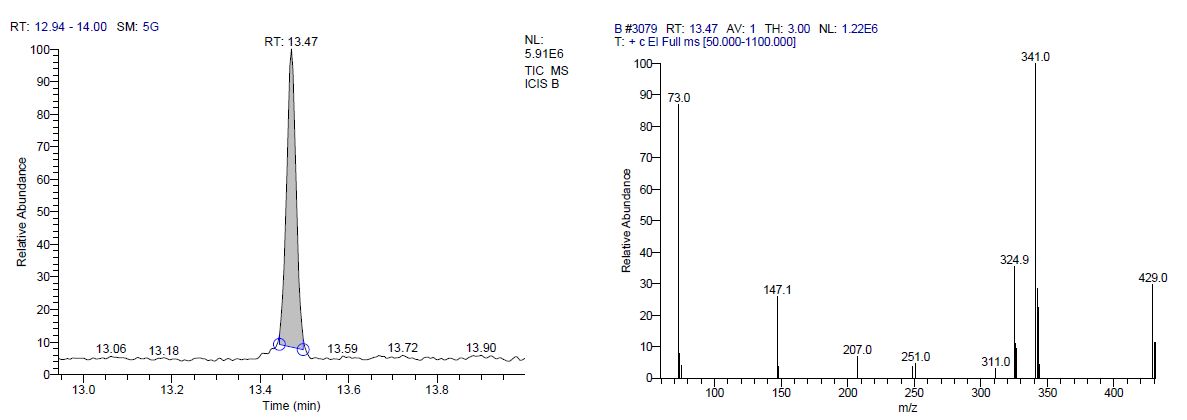


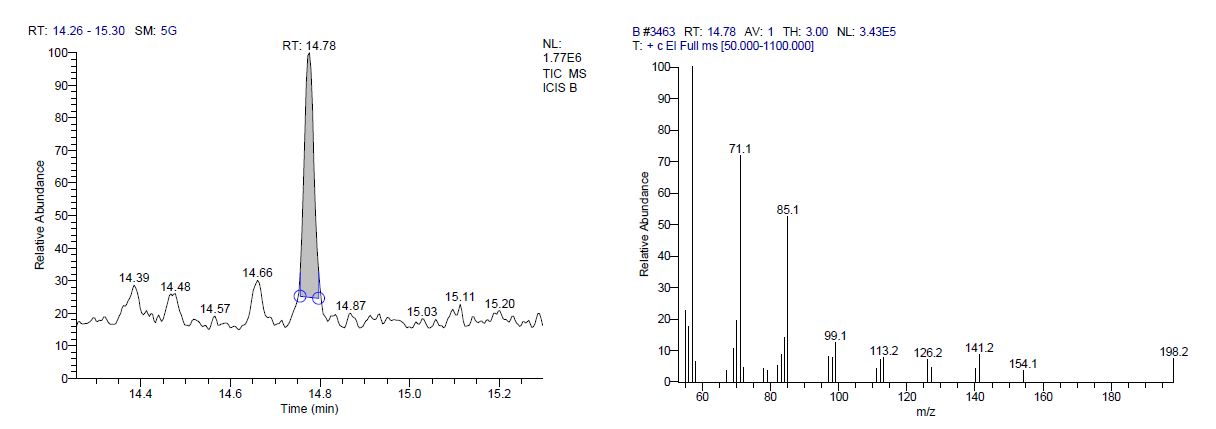


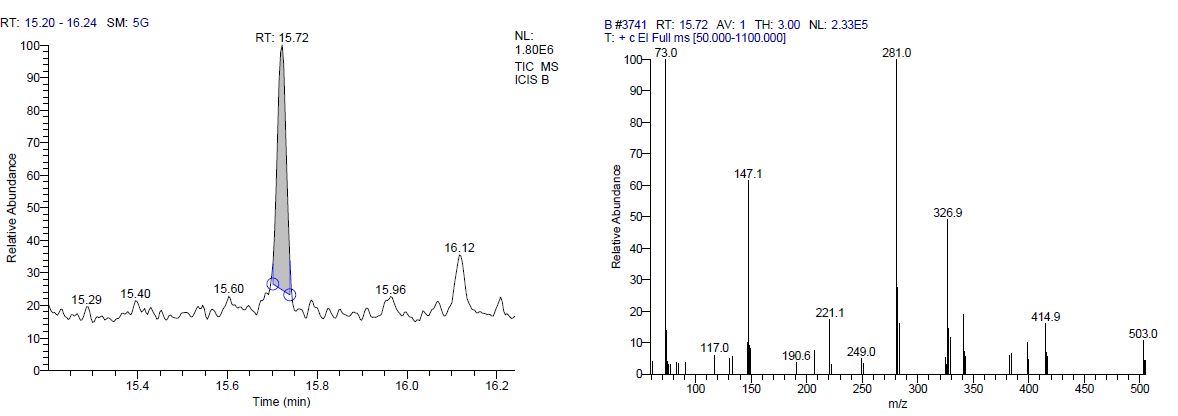


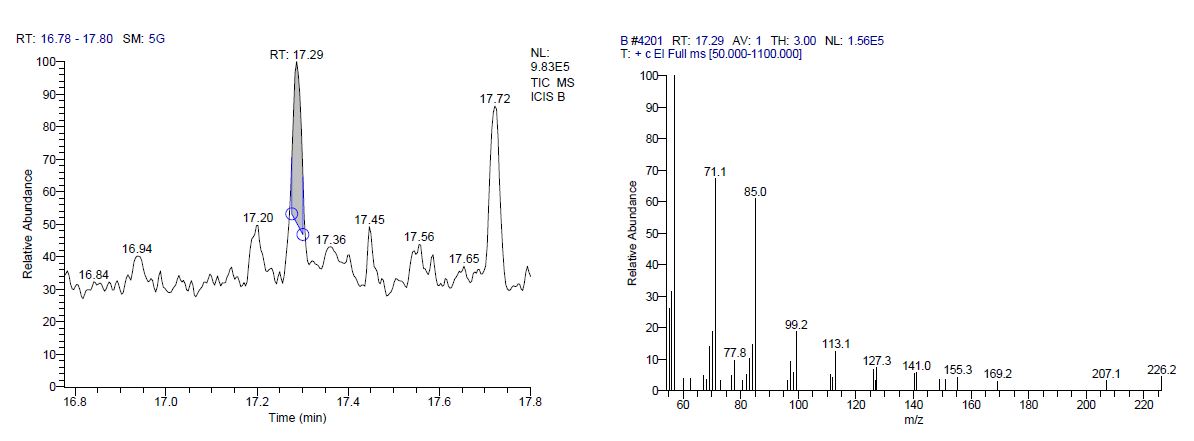


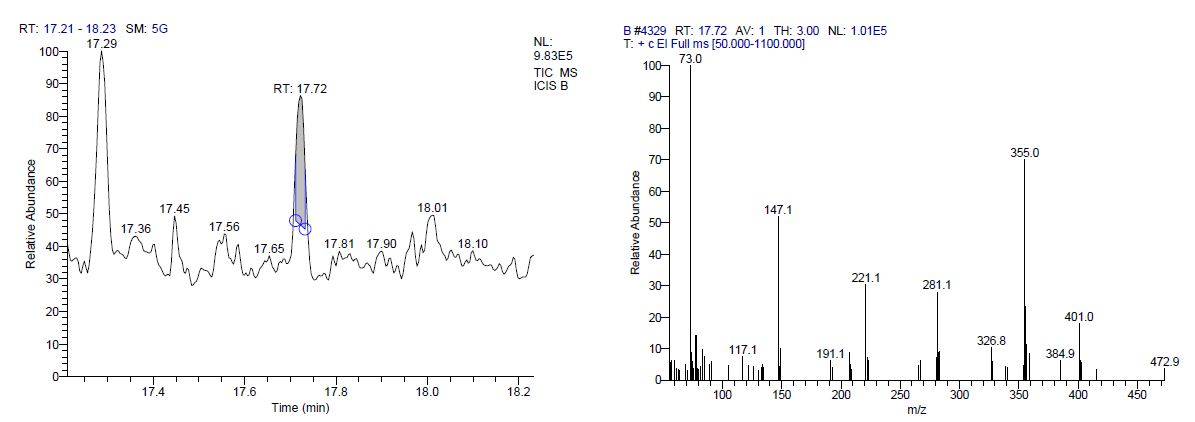


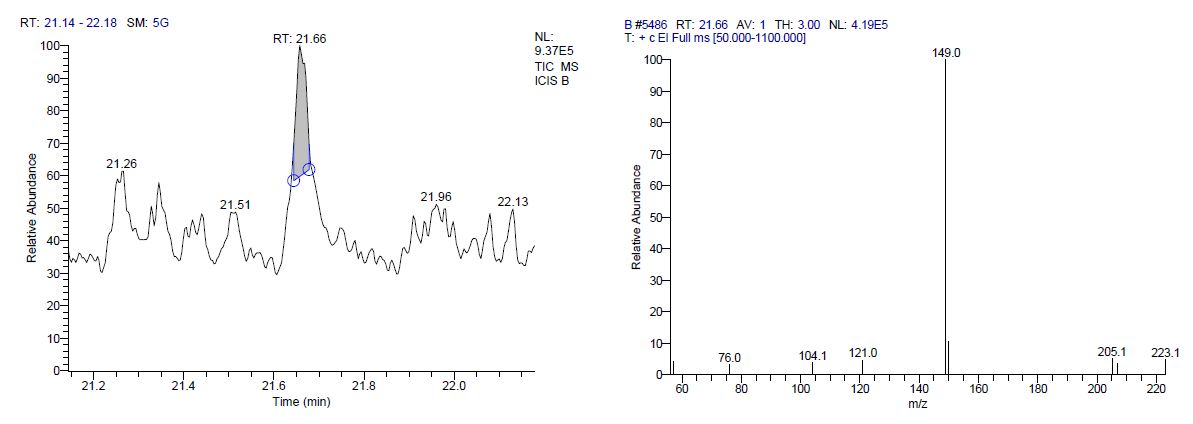


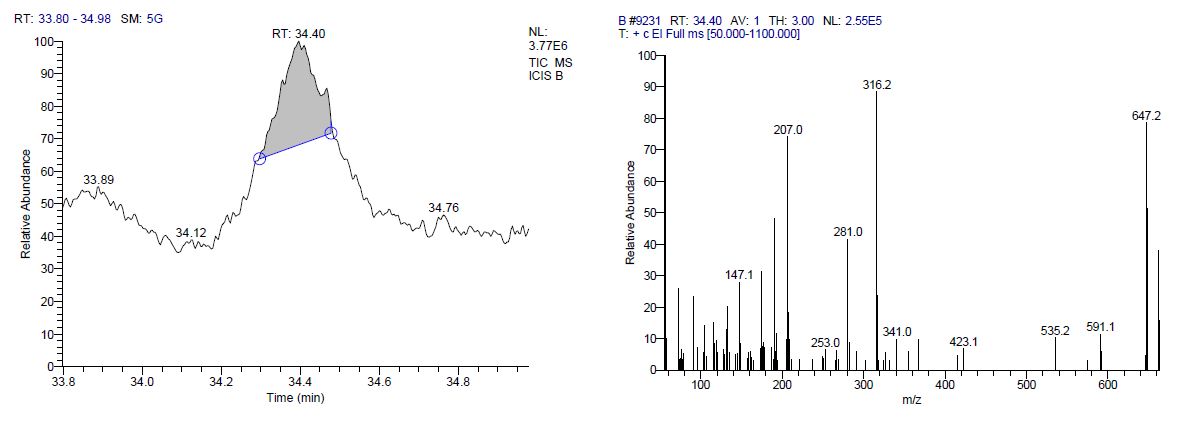

Supplement: Supplementary file 6 — Additional file 6: GC mass report of the compounds detected in band S3 of flavonoids from Symplocos racemosa bark. [file 40360_2020_453_MOESM6_ESM.docx]
